# Global Plant Stress Signaling: Reactive Oxygen Species at the Cross-Road

**DOI:** 10.3389/fpls.2016.00187

**Published:** 2016-02-23

**Authors:** Nasser Sewelam, Kemal Kazan, Peer M. Schenk

**Affiliations:** ^1^Botany Department, Faculty of Science, Tanta UniversityTanta, Egypt; ^2^Commonwealth Scientific and Industrial Research Organization Agriculture, Queensland Bioscience Precinct, St LuciaQLD, Australia; ^3^Queensland Alliance for Agriculture & Food Innovation, The University of Queensland, BrisbaneQLD, Australia; ^4^Plant-Microbe Interactions Laboratory, School of Agriculture and Food Sciences, The University of Queensland, BrisbaneQLD, Australia

**Keywords:** abiotic stress, biotic stress, oxidative stress, plant defense, plant stress signaling, reactive oxygen species

## Abstract

Current technologies have changed biology into a data-intensive field and significantly increased our understanding of signal transduction pathways in plants. However, global defense signaling networks in plants have not been established yet. Considering the apparent intricate nature of signaling mechanisms in plants (due to their sessile nature), studying the points at which different signaling pathways converge, rather than the branches, represents a good start to unravel global plant signaling networks. In this regard, growing evidence shows that the generation of reactive oxygen species (ROS) is one of the most common plant responses to different stresses, representing a point at which various signaling pathways come together. In this review, the complex nature of plant stress signaling networks will be discussed. An emphasis on different signaling players with a specific attention to ROS as the primary source of the signaling battery in plants will be presented. The interactions between ROS and other signaling components, e.g., calcium, redox homeostasis, membranes, G-proteins, MAPKs, plant hormones, and transcription factors will be assessed. A better understanding of the vital roles ROS are playing in plant signaling would help innovate new strategies to improve plant productivity under the circumstances of the increasing severity of environmental conditions and the high demand of food and energy worldwide.

## Introduction

Plants are increasingly subjected to a variety of environmental stresses which diminish the productivity of various economically important crops. Every year, the world loses a huge amount of crop production through scarcity of water, extreme temperatures, high soil salinity, herbivore attack, and pathogen infection.

The sessile nature of plants has resulted in the evolution of complicated protection mechanisms to survive different environmental challenges. One of the stress tolerance mechanisms is the ability to sense complex stress factors and respond appropriately. Activation of complex signaling pathways helps plants to achieve this. To better understand plant signaling pathways would enable us to modify plants to improve their adaptability. However, this requires reducing the complexity associated with signaling pathways. Focusing on the points at which different signaling pathways converge, rather than studying the branches of these pathways, would be helpful as a starting point. The rapid generation of reactive oxygen species (ROS) represents a common plant response to different biotic and abiotic stresses (Lamb and Dixon, 1997; Orozco-Cardenas and Ryan, 1999; Kovtun et al., 2000; Kotchoni and Gachomo, 2006; Mittler et al., 2011; Petrov and Van Breusegem, 2012; Noctor et al., 2014; Xia et al., 2015) and thus a basis to unify signaling events.

Recent genomic technologies, especially global gene expression tools, have not only produced new details about plant signaling pathways but also raised many historical questions including the followings: Is there a specific linear signaling pathway for each stress? If so, what about the observed cross-talk? Is there a big common signaling network from which many branches arise for specificity? If so, what about the different receptors? What do represent the points at which different branches of signaling pathways converge? If ROS are at the points of integrating signaling outputs from different signaling pathways, then what are the ROS receptors? What are the upstream and downstream signaling components of ROS? How do ROS set signaling specificity? What about the photosynthetic machinery that generates ROS; does it and its ROS and redox system represent a primary source of the plant signaling battery? As discussed throughout this review, most of these questions have been answered (or are being answered) while we will be in a better shape in providing more definite answers to the remaining ones in the near future.

This review presents a discussion about these historical questions by considering the so complex nature of plant stress signaling networks. A special attention will be given on reviewing signaling players and events such as receptors/sensors, secondary messengers, specificity, cross-talk, redundancy, feedback regulations, alternative promoter usage, alternative splicing, nucleo-cytoplasmic trafficking, and epigenetics. Here, an attempt will also be made to indicate the kinds of studies required to fill in the gaps. A specific thought to photosynthetic activities and ROS as the primary source of the signaling battery in plants will be presented. As ROS production represents a common plant response to almost all environmental challenges, a special emphasis will be devoted here to ROS production, scavenging, damaging effects, signaling roles and how they work upstream and downstream of other signaling components, e.g., calcium, redox homeostasis, membranes, G-proteins, MAPKs, plant hormones [such as salicylic acid (SA), jasmonic acid (JA), abscisic acid (ABA), and ethylene] and transcription factors (TFs). We hope to present a holistic summary of various signaling components and concepts that are important for a plant biologist to take into consideration when analyzing signaling events involved in plant response to the ever changing environment. This understanding would help construct comprehensive signaling networks which in turn innovate new strategies to improve plant productivity under the increasing severity of environmental stress conditions and the high global demand for food and energy.

## Plant Signaling Networks

### Signaling Networks are Complex

Recently, our knowledge about signaling mechanisms in plants starting from stimulus sensing to final response has increased. It is obvious that there is a large number of components underlying signaling mechanisms, including a high degree of interconnectivity, many spatio-temporal levels, and a complicated tune of signal transduction pathways. For example, the changes at the expression of certain genes under a definite environmental condition are not necessarily translated into metabolic and structural changes where the interactions between various aspects, including post-transcriptional and post-translational modifications, compartmentalization, metabolite stability, substrate availability may lead to an unexpected response (Krasensky and Jonak, 2012).

**FIGURE 1:**
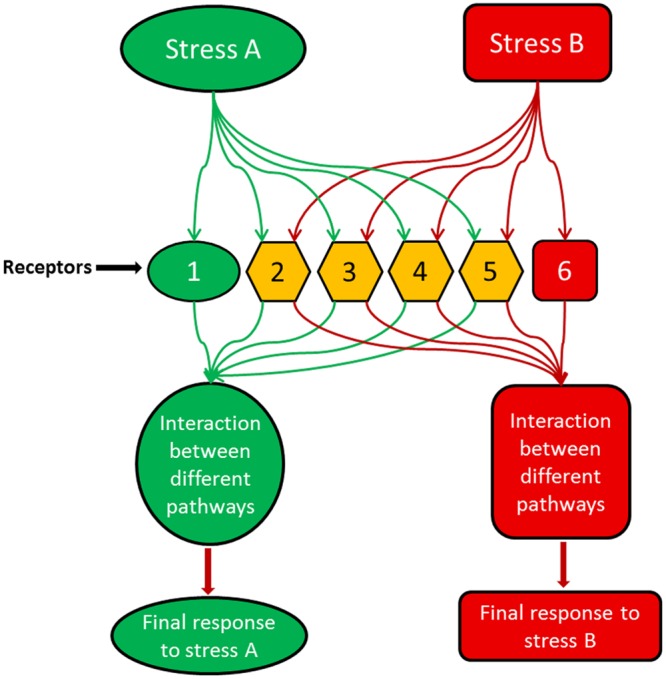
**A model illustrating how different stresses or stimuli could activate overlapping receptors/sensors but produce distinct final outputs which are specific to each stimulus.** In the model, stress A activates different receptors, e.g., 1, 2, 3, 4 and 5, while stress B is perceived by receptors 2, 3, 4, 5, and 6. Receptor 1 is activated only by stress A, while receptor 6 is activated only by stress B. The other receptors are shared between both stimuli representing the cross-talk between stress A and B. With stress A, the interaction between the downstream signaling events led by the receptor combination of 1, 2, 3, 4, and 5 produce a final output which can be completely different from the outcome of the receptor combination of 2, 3, 4, 5, and 6 with stress B.

Moreover, it is becoming increasingly clear that signaling networks are not linear; rather they are part of a complicated and dynamic network with substantial overlap among their branches (Knight and Knight, 2001). Accordingly, rather than one sensor, there are many sensors that perceive certain stress conditions and control all downstream signals (**Figure 1**). Each sensor controls a branch of the signaling pathway activated by one aspect of the stress condition. For instance, temperature stress is well-known to change the physical state (fluidity) of membranes (Murata and Los, 1997; Königshofer et al., 2008), but this may not be the only condition that elicits signaling events under this stress. Changes of the conformation/activity of some intracellular proteins may also be involved in signaling to cold stress. Therefore, it is likely that the initial stress signal is perceived by multiple primary sensors, and then a cascade of signaling events is initiated by secondary signals such as plant hormones and calcium, which differ from the primary signal in time (coming late) and space (different compartments). Also, these secondary signals may differ in specificity from primary stimuli, may be shared by various stress pathways, and may underlie the interaction among signaling pathways for different insults and stress cross-protection (**Figure 1**). Consequently, multiple signaling mechanisms may be activated by one stimulus/stress initiating pathways differ in time, space, and outputs. Using shared signaling intermediates, such as phytohormones, these pathways may interconnect or interact with one another producing an intertwined signaling network (Xiong et al., 2002).

**FIGURE 2:**
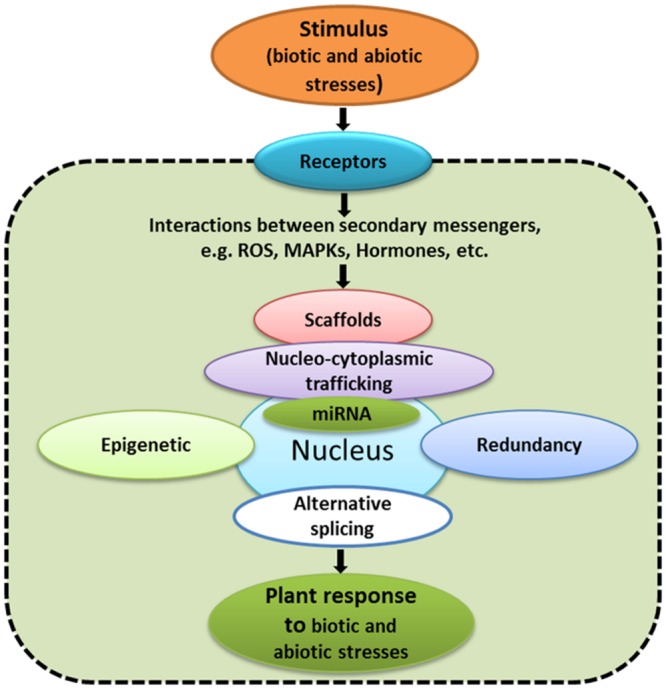
**Examples of aspects that have to be taken into consideration while studying signaling networks**.

The changes in gene expression do not represent end points for signaling pathways. There are many other aspects, including transcriptional, post-transcriptional and post-translational regulation, redundancy, alternative promoter usage, alternative splicing, protein trafficking, non-coding RNA, and epigenetic effects (**Figure 2**) that regulate signaling pathways. In the following sections, these latter aspects will be briefly discussed.

### Redundancy and Signaling

During stress, plant signaling networks have a high ability to compensate the effects of disturbances in neighboring nodes and related signaling pathways (Chiwocha et al., 2005). The simple explanation for functional redundancy may be brought about by duplicate genes that eukaryotic genomes contain (Stelling et al., 2004; De Smet and Van de Peer, 2012). It was reported that about one-quarter of functional redundancy in *Saccharomyces cerevisiae* can be explained by compensation by duplicate genes (Gu et al., 2003). However, while sequence redundancy explains some functional redundancy, the ability of networks to compensate the effects of perturbations in neighboring nodes and related pathways could be the main cause (Chiwocha et al., 2005). Of course, this redundancy represents a great advantage to the organism to cope with the ever changing environment. However, this represents a big problem to scientists who are studying signaling transduction pathways, especially when using knockout mutants. They usually prefer an altered phenotype after modulation of single genes (Chiwocha et al., 2005). Therefore, it is imperative, while studying a signaling pathway, to take into consideration the fact that many signaling components can be functionally redundant under a given experimental condition or current phenotypic methods are not sensitive enough to detect the consequences of knocking out single genes. Therefore, gene knockouts should be examined under a variety of experimental conditions, using genome-wide gene expression profiling and other omic technologies wherever possible.

### Alternative Promoter Usage, Alternative Splicing, and Signaling

Many eukaryotic genes have multiple promoter elements. Each one is subjected to different regulatory factors under different situations. Alternative promoter usage is greatly linked to alternative splicing of internal exons and often has physiological implications (Kornblihtt, 2005). Alternative splicing produces multiple transcripts from the same gene and potentially different proteins. In turn, it represents a key post-transcriptional regulatory mechanism for expanding proteomic diversity and functional complexity in higher eukaryotes (Reddy, 2007; Carvalho et al., 2012). At the level of post-transcriptional mRNA processing, alternative splicing represents the primary mechanism to control the number of intracellular components (Bardo et al., 2002). There is substantial evidence that cellular signaling networks control the number and types of network components using alternative splicing. In the human genome, it was reported that 40–60% of the genes are subjected to alternative-splicing, with estimates of an average of 8 exons per gene (Thanaraj, 2004). In plants, alternative splicing has received less attention because this phenomenon was rare to be considered (Reddy, 2007). Others reported that, in plants, alternative splicing is ubiquitous and can mediate a bounty of transcriptome and proteome complexity (Kazan, 2003; Wang and Brendel, 2006). In the model plant *Arabidopsis*, 4,707 genes showed 8,264 alternative splicing events (Wang and Brendel, 2006). It was reported that alternative splicing of introns is involved in the regulation of the kinase activity of the MIK GCK-like MAP4K. Four different mature mRNAs of MIK were found to be accumulated with different expression profiles during maize development (Castells et al., 2006). Under stress conditions alternative splicing of pre-mRNAs dramatically increases (Reddy, 2007). Virus infection led to multiple novel intron-retaining splice variants in *Brachypodium distachyon* (Mandadi et al., 2015). So, while studying signaling networks, it is important to consider not only the signaling events leading to transcriptional changes, but also protein modifications (such as phosphorylation and glycosylation).

### Protein Trafficking and Signaling

The nuclear envelope separates the nuclear compartment containing the genes from the cytoplasm where mRNA translation and protein synthesis occurs. Therefore, all nuclear proteins, including TFs, must be imported to the nucleus. This nucleo-cytoplasmic trafficking is under complex control. The *Arabidopsis* genome, for example, contains at least 17 genes encoding importin B-like nuclear transport receptors (Bollman et al., 2003). TFs and kinases are the main regulatory components in almost all signaling pathways. Hence, it is important, while studying signal transduction pathways, to consider not only the signaling events modulating the expression of regulatory genes and proteins and their downstream interactors, but also to think about how the access of these TFs to the target genes is regulated. In general, control of transcription on both the level of TF activity and the level of nucleo-cytoplasmic partitioning are combined to create a redundant network of regulatory switches to orchestrate different signaling mechanisms (Merkle, 2004; Parry, 2015). During pathogen infection, recent reports have suggested the involvement of the nucleo-cyctoplasmic trafficking of plant R proteins to achieve effector-triggered immunity and mediate disease resistance (Shen et al., 2007; Liu and Coaker, 2008). For more intensive discussion on nucleo-cytoplasmic trafficking and signaling, see Merkle (2004) and Parry (2015).

### MicroRNA and Signaling

At the post-transcriptional level, microRNAs (miRNAs) are a class of small non-coding RNAs that are increasingly being recognized as key modulators of gene expression (Covarrubias and Reyes, 2010; van Rooij, 2011; Ding et al., 2013, 2015). miRNAs regulate the expression of relevant genes by binding to reverse complementary sequences, resulting in cleavage or translational inhibition of the target RNAs (Khraiwesh et al., 2012). miRNAs are reported to play important roles in biotic and abiotic stress responses in plants. Through repressing the expression of the respective target genes encoding regulatory and functional proteins, various miRNAs were reported to play crucial roles in drought stress responses, including ABA response, osmoprotection, and antioxidant defense (Ding et al., 2013). It was reported that H_2_O_2_ stress led to differential expression of seven miRNA families. The targets of these H_2_O_2_-responsive miRNAs were found to be involved in different cellular responses and metabolic processes including transcriptional regulation, nutrient transport, and programmed cell death (PCD; Li et al., 2011). The downregulation of miR398 was found to mediate post-transcriptional induction of two Cu/Zn superoxide dismutase (SOD) genes and be important for oxidative stress tolerance in *Arabidopsis* (Sunkar et al., 2006). During biotic stress, miRNAs were found to contribute to antibacterial resistance of *Arabidopsis* against *Pseudomonas syringae* via repressing auxin signaling (Navarro et al., 2006).

### Epigenetic Effects and Signaling

Epigenetics (the study of heritable changes in gene expression that are not due to changes in DNA sequence; Bird, 2007) has become one of the hottest subjects of research in plant functional genomics since it plays an important role in developmental gene regulation, response to environmental stresses, and in natural variation of gene expression levels (Chinnusamy and Zhu, 2009; Sahu et al., 2013; Springer, 2013). Epigenetic effects are ascribed to a variety of molecular mechanisms including stable changes in protein structure, expression of small RNAs, and chromatin modifications. Chromatin modifications include DNA methylation, histone variants, remodeling of chromatin structure, and modification of histones including acetylation, methylation, ubiquitination, and phosphorylation (Springer, 2013). These mechanisms have the ability to regulate almost all genetic functions, including replication, DNA repair, gene transcription, gene transposition, and cell differentiation. For example, modifications in chromatin and generation of small RNAs have been shown to be involved in transcriptional and post-transcriptional control of gene expression during stress responses in plants (Madlung and Comai, 2004; Angers et al., 2010). These modifications are tissue-, species-, organelle-, and age-specific (Vanyushin and Ashapkin, 2011). The changes in hormonal levels that occur during biotic and abiotic stresses can control DNA methylation and other epigenetic effects (Zhang et al., 2012) resulting in plant adaptation (Mirouze and Paszkowski, 2011). Consequently, decoding how epigenetic mechanisms work in developmental gene regulation and during plant response to the environmental stresses is important. In turn, deciphering these mechanisms will also provide valuable information for potential applications, including genetic manipulation of plants toward enhanced tolerance to environmental stresses (Sahu et al., 2013; Springer, 2013).

### Construction of Ever-Larger Signaling Networks is an Urgent Task

Indeed, cellular, genetic, genomic, proteomic, and metabolomic data platforms have resulted in increasingly more detailed descriptions of signaling mechanisms, which have raised the necessity for construction of ever-larger signaling networks (Papin et al., 2005; Baginsky et al., 2010). Understanding the function of these signaling networks through reconstructing the available data about signaling pathways is crucial for studying plant’s responses to different diseases and stresses.

Therefore, it is important to somewhat simplify this complexity. The start could be at the points at which the different signaling pathways converge, rather than studying the branches. Consequently, studying the phenomenon of cross-talk may represent a good point to start to unravel global signaling networks. Additionally, tolerance across different stresses is extremely important for agriculture where plants with tolerance to more than one stress can be produced through breeding as well as transformation (Sewelam et al., 2014b). It was stated that, although different environmental challenges use unique mechanisms to initiate their specific responses, all forms of stresses seem to induce a common set of responses (Levitt, 1972). More recently, it was reported that different stress-induced changes in gene and protein expression include similar fingerprints under various environmental insults in different organisms (Desikan et al., 2001; Scandalios, 2002; Laloi et al., 2004; Polidoros et al., 2005; Walley and Dehesh, 2009; Baena-González, 2010; Atkinson and Urwin, 2012). In this regard, it was found that the accelerated generation of ROS is a common plant response to different biotic and abiotic stresses (Allen et al., 1995; Goulet et al., 1997; Noctor, 1998; Orozco-Cardenas and Ryan, 1999; Asai et al., 2000; Miller et al., 2010; Petrov and Van Breusegem, 2012; Noctor et al., 2014; Perez and Brown, 2014; Hossain et al., 2015; Xia et al., 2015). The remainder of this review will be devoted for studying ROS signaling. ROS production, scavenging, damaging effects, signaling roles and how ROS work upstream or downstream of other signaling components will be discussed.

## Reactive Oxygen Species at the Cross-Road

**FIGURE 3:**
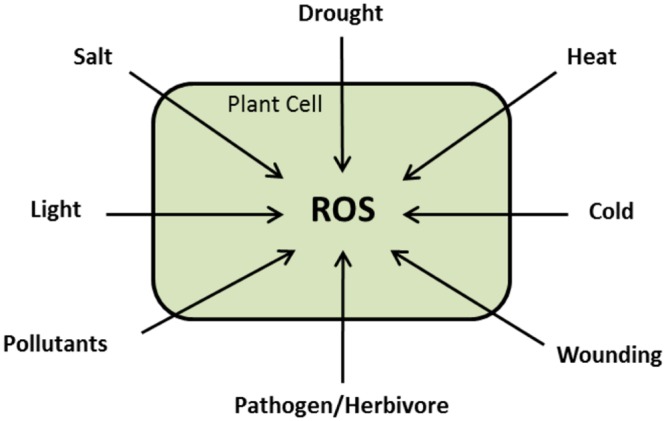
**Schematic presentation showing that ROS are versatile signaling molecules during plant response to different stresses**.

During normal growth and development, ROS are produced in different cellular compartments in living cells with increased production under biotic and abiotic challenges (**Figure 3**; Møller et al., 2007; Miller et al., 2010). The traditional notion that ROS are toxic by-products of plant metabolism has changed. Substantial experimental data are available assuring that ROS are highly controlled signaling molecules able to transfer the environmental signals, with other signaling intermediates, to the genetic machinery (Polidoros et al., 2005). Here, we present a summary about ROS chemistry and signaling that would help understanding of the next sections. For detailed descriptions, we suggest the following reviews; Mittler (2002), Apel and Hirt (2004), Laloi et al. (2004), Mittler et al. (2004, 2011), Asada (2006), Halliwell (2006), Møller et al. (2007), Heller and Tudzynski (2011), Wrzaczek et al. (2013), Baxter et al. (2014).

### Definition and Chemistry of ROS

**FIGURE 4:**
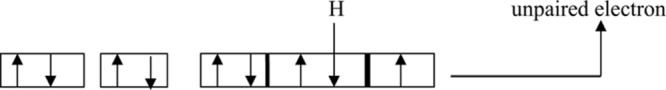
**Hydroxyl radical (OH^∙^) as an example for ROS**.

Molecular oxygen, in its ground state, is relatively unreactive. Nevertheless, during normal metabolic activity, and as a result of various environmental stresses, O_2_ is capable of giving rise to dangerous reactive states such as free radicals (Polidoros et al., 2005; Phaniendra et al., 2015). Reactive oxygen intermediates may result from the excitation of O_2_ to form singlet oxygen (^1^O_2_; Triantaphylidès and Havaux, 2009) or from the transfer of one, two, or three electrons to O_2_ to form, respectively, a superoxide radical ($O_{2}^{•-}$), H_2_O_2_ or a hydroxyl radical (OH^∙^; Mittler, 2002). The free radical might be defined as any species capable of an independent existence that contains one or more unpaired electrons; an unpaired electron being one that is alone in an orbital (Halliwell, 1991; **Figure 4**).

Radicals are generally more reactive than non-radicals because electrons are more stable when paired together in orbitals, but when an electron occupies an orbital by itself it has two possible directions of spin. On the other hand, H_2_O_2_ and singlet oxygen, themselves, can be quite toxic to cells although they are non-radicals. Accordingly, the term ROS has been introduced to describe collectively, not only free radicals but also other toxic non-radicals (Halliwell, 1991).

### ROS Production *In vivo*

Reactive oxygen species are generated during normal metabolic processes. In addition, they are produced as an inevitable result of electron transport chains in chloroplast and mitochondria. As a result, electrons fall onto O_2_, generating different ROS. Furthermore, abiotic and biotic stresses can further exaggerate the production and accumulation of ROS (Bhattacharjee, 2005). Mittler (2002) mentioned ten sources for production of ROS in plant cells, including, in addition to photosynthetic and respiratory electron transport chains, NADPH oxidase, photorespiration, amine oxidase, and cell wall-bound peroxidases. In chloroplasts, for example, ROS can be produced at photosystem I (PSI) as well as at PSII. During stress conditions the absorbed light energy exceeds the capacity of photosynthesis to use it through photosynthetic electron transport. As a result, various ROS are formed, including singlet oxygen (^1^O_2_) at PSII and superoxide radicals ($O_{2}^{•-}$) at PSI and PSII as byproducts (Pospisil et al., 2004; Asada, 2006; Schmitt et al., 2014). At PSII, the excess energy may be transferred from excited chlorophyll to molecular oxygen (energy is transferred not electrons) forming ^1^O_2_ as indicated below;

$$\mathrm{Pigment}+\mathrm{light}\to \mathrm{excited}\;\mathrm{pigment}\;(\mathrm{P*})\;\mathrm{P*+}\;O_{2}\to {\mathrm{P+}}^{1}O_{2}$$

Reaction (1)

Under certain conditions (when the transport of photosynthetic products out of the chloroplast or the re-oxidation of NADPH is inhibited as occurs during different stresses) and O_2_ reduction (electron transfer) at PSI, superoxide radical formation takes place (Furbank et al., 1983). Then these $O_{2}^{•-}$ radicals are dismutated into H_2_O_2_ spontaneously as well as through the action of SOD. Later on, inside the chloroplast, $O_{2}^{•-}$ and H_2_O_2_ can react with each other in the presence of soluble metal ions, such as iron, to form the more reactive hydroxyl radicals according to the Haber–Weiss reaction (Bowler et al., 1992).

$$H_{2}O_{2}+O_{2}^{•-}\overset{{\mathrm{Fe}}^{\mathrm{2+}}{\mathrm{/Fe}}^{\mathrm{3+}}}{⟶}{\mathrm{OH}}^{•}+O_{2}+{\mathrm{OH}}^{-}$$

Reaction (2)

On the other hand, mitochondria represent a main source for ROS generation in aerobic organisms. It was estimated that from 1 to 5% of the oxygen taken up by isolated mitochondria is used in ROS production (Møller et al., 2007). The complete reduction of O_2_ to water through the respiratory electron transport chain requires four electrons.

$$O_{2}+4e^{-}+4H^{+}\to 2H_{2}O$$

Reaction (3)

But, as a consequence of spin restrictions, O_2_ cannot accept the four electrons at once, but one at a time. As a result, during O_2_ reduction, stable ROS intermediates such as $O_{2}^{•-}$, H_2_O_2_, and OH^∙^ are formed in a stepwise fashion as follow;

$$\begin{array}{l} O_{2}+e^{-}\to O_{2}^{•-}\\ O_{2}^{•-}+e^{-}+2H^{+}\to H_{2}O_{2}\\ H_{2}O_{2}+e^{-}+H^{+}\to H_{2}O+{\mathrm{OH}}^{•}\\ {\mathrm{OH}}^{•}+e^{-}+H^{+}\to H_{2}O \end{array}$$

Reaction (4)

In peroxisomes, H_2_O_2_ is produced during the process of photorespiration via the action of the enzyme glycolate oxidase (Møller et al., 2007). Also, the plasma membrane-bound NADPH oxidases make a big contribution to ROS production in plant cells, especially during pathogen infections (Torres and Dangl, 2005).

### Damaging Effects and Scavenging of ROS

Plants are well-adapted for minimizing the damage that could be induced by ROS under natural growth conditions. However, O_2_ toxicity emerges when the production of ROS exceeds the quenching capacity of the protective systems due to stress conditions (Bowler et al., 1992; Yuasa et al., 2001; Miller et al., 2009; Akter et al., 2015). As a consequence, different ROS, including $O_{2}^{•-}$, H_2_O_2_, OH^∙^, and singlet oxygen, are formed, leading to oxidizing and destroying lipids, proteins, and DNA in the stressed cells (for intense information on this topic see Scandalios, 2005; Møller et al., 2007; Vanderauwera et al., 2011). Thus, plant cells have evolved antioxidant mechanisms to combat the danger posed by the presence of ROS (Baxter et al., 2007; Gill and Tuteja, 2010; Miller et al., 2010; Heller and Tudzynski, 2011; Wrzaczek et al., 2013; Schmitt et al., 2014). Mittler (2002) has reported the presence of ten mechanisms to remove ROS, in addition to five ways to avoid ROS production in plant cells. These include several enzymatic and non-enzymatic mechanisms. The enzymatic mechanisms include antioxidant enzymes, such as SOD (which converts $O_{2}^{•-}$ to H_2_O_2_), catalases and peroxidases (which remove H_2_O_2_). The non-enzymatic mechanisms of ROS removal include antioxidant molecules, such as ascorbic acid, glutathione, carotenoids, and α-tocopherol (Noctor, 1998; Asada, 1999; Mittler, 2002). It was reported that there is a network of 152 genes involved in managing the level of ROS in *Arabidopsis* (Mittler et al., 2004).

In addition to these antioxidant mechanisms which scavenge the already formed ROS, plants have evolved smart ways to avoid the production of toxic forms of oxygen. These avoiding mechanisms include anatomical adaptations, such as leaf movement and curling, C4 or CAM (Crassulacean Acid Metabolism), chlorophyll movement, suppression of photosynthesis, and photosystems and antenna modulators (Maxwell et al., 1999; Mittler, 2002).

### ROS Signaling and Specificity

For a signaling molecule to be effective, it needs to be produced quickly and efficiently on demand, to induce distinct effects within the cell, and to be removed rapidly and efficiently when no longer required (Neill et al., 2003). ROS are produced instantly after the onset of the stress. In addition, ROS are very reactive; they can react with membrane lipids, carbohydrates, proteins and DNA. ROS such as H_2_O_2_ can defuse through the biological membranes through aquaporins (Bienert et al., 2007; D’Autreaux and Toledano, 2007; Dynowski et al., 2008; Mubarakshina et al., 2010; Borisova et al., 2012) leading to systemic responses. Moreover, living cells have very efficient antioxidant systems, including enzymatic and non-enzymatic mechanisms, to put ROS under a precise control (Foyer and Noctor, 2005). Collectively, all of these features of ROS render them ideal signaling components.

Levine et al. (1994) have suggested a signaling role for H_2_O_2_, controlling the hypersensitive response and promoting the expression of glutathione-*S*-transferase and glutathione peroxidase encoding genes. Many studies have suggested signaling roles for ROS in developmental processes as well as biotic and abiotic responses (Apel and Hirt, 2004; Foyer and Noctor, 2005; Gadjev et al., 2006; Miller et al., 2009; Mittler et al., 2011; Wrzaczek et al., 2013; Perez and Brown, 2014). In an early study, the genomic response of *Escherichia coli* cells to H_2_O_2_ treatment was examined with a DNA microarray composed of 4169 open reading frames (Li et al., 2001). In this study, the mRNA of 140 genes (in wild-type) was considerably induced after H_2_O_2_ treatment. On exposure of *S. cerevisiae* cells to H_2_O_2_, expression of about one-third of all yeast genes had changed suggesting that ROS can cause massive alterations in the biology of the oxidative-stressed cells (Gasch et al., 2000). Using cDNA microarray technology from a sample of 11,000 expressed sequence tags (ESTs), 175 non-redundant EST were identified that are regulated by H_2_O_2_ in *Arabidopsis* (Desikan et al., 2001).

To this end, it is quite evident that ROS operate as intracellular signaling molecules, but how they can set specific signaling duties is still controversial. This controversy arises from what seems to be a paradox between the reactive nature of ROS that renders them indiscriminate and the specificity that is required for signaling (D’Autreaux and Toledano, 2007). In general, the specificity in signaling pathways is mediated via the non-covalent binding of a ligand to its cognate receptor through a shape-complementary fit between macromolecules. On the other side, ROS deliver signaling events via chemical reactions with specific atoms, such as iron (Fe) and sulphur (S), of target proteins that lead to protein modifications (Nathan, 2003). ROS can also react with different target proteins whenever the chemical reaction is possible. The remaining question is how specificity in ROS signaling is managed? By looking into the chemical characteristics and the biological activities of each ROS, including $O_{2}^{•-}$, H_2_O_2_, OH^∙^ and singlet oxygen (^1^O_2_), an answer to this question could be revealed.

$O_{2}^{•-}$ is a by-product of electron transport chains of photosynthesis and respiration and is produced by NADPH oxidases and cell wall peroxidases. In *E. coli*, the steady-state concentration of $O_{2}^{•-}$ is very low (∼10^-11^ M; Halliwell and Gutteridge, 1999), which reflects its instability; this is mainly due to spontaneous and SOD-mediated $O_{2}^{•-}$ dismutation to H_2_O_2_. The instability of $O_{2}^{•-}$ and its inability to diffuse through membranes because of its negative charge make this ROS relatively poor signaling molecule. However, due to high attraction, $O_{2}^{•-}$ oxidizes Fe–S clusters at a rate that is almost diffusion limited (Storz et al., 1990; Storz and Imlayt, 1999).

**H_2_O_2_** is actually a poor oxidant and reacts mildly with [Fe–S] (rate constant of 10^2^–10^3^ M^-1^ s^-1^), loosely bound metals and, very slowly, with glutathione and free cysteine (Cys) (Imlay, 2003). By contrast, its reactivity toward Cys residues can significantly increase to 10–10^6^ M^-1^ s^-1^. H_2_O_2_ is relatively stable (cellular half-life ∼1 ms, steady-state levels ∼10^-7^ M; D’Autreaux and Toledano, 2007), and can diffuse through biological membranes because it is not charged. Its selective reactivity, stability and diffusability make H_2_O_2_ fit for signaling. As a second messenger, H_2_O_2_ can mediate intracellular signal transduction through chemoselective oxidation of Cys residues in signaling proteins, such as glutathione, thioredoxins, and peroxiredoxins (Paulsen and Carroll, 2010).

OH^∙^ is the most highly toxic ROS. It has high indiscriminate reactivity, which limits its diffusion to sites of production (half-life 10^-9^ s; Halliwell and Gutteridge, 1999), even though OH^∙^ seems to operate in H_2_O_2_ sensing (D’Autreaux and Toledano, 2007).

Singlet oxygen (**^1^O_2_**) is an excited state molecule. The half-life time of ^1^O_2_ is very short (∼100 ns) and it can travel only a very short distance in cells (<100 nm; Moan, 1990; Niedre et al., 2002). This could be because it reacts very rapidly with amino acids, unsaturated lipids, and other cell constituents. As a result, ^1^O_2_ can react directly only with molecules in close proximity to its production location, i.e., in the chloroplast (Kochevar, 2004; Triantaphylidès and Havaux, 2009). This means that ^1^O_2_ could deliver specific signaling events mainly through spatial aspects of ROS production.

In addition to the previous chemical characteristics that render ROS able to set specificity as signaling molecules, a non-ROS intermediate in a ROS signaling pathway can regulate additional pathways that are physically non-adjacent to the pathway in which it was formed (Nathan, 2003). For instance, a ROS that is produced in a cellular compartment could specifically activate a secondary messenger such as a MAPK or a plant hormone, which in turn activate remote signaling pathways. In *Arabidopsis*, it was suggested that histidine kinase ethylene receptor ETR1 is important for H_2_O_2_ perception during stomatal closure (Bright et al., 2006). In addition, it was reported that indirect activation of TFs by ROS may be mediated by some members of MAPK cascades (Asai et al., 2000). Interestingly, it was shown that H_2_O_2_ originating in different subcellular sites induces different responses. H_2_O_2_ produced in chloroplasts was found to activate early signaling responses, including TFs and biosynthetic genes involved in production of secondary signaling messengers; while H_2_O_2_ produced in peroxisomes was found to induce transcripts involved in protein repair responses (Sewelam et al., 2014a). Moreover, ROS-mediated changes in the cellular redox homeostasis could set highly specific signaling roles for ROS. For example, different pathways could sense and weigh the change in cellular redox balance resulting from the change of intracellular ROS concentration, then translate these changes into highly specific cellular signals that direct the cell to produce a relevant adaptive response (Foyer and Noctor, 2005). In simple organisms, such as bacteria and yeast, the enhanced production of ROS is perceived by change in redox homeostasis which in turn is delivered to redox sensitive TFs (Costa and Moradas-Ferreira, 2001; Georgiou, 2002). In addition, it has been proposed that ROS may be perceived indirectly by sensing changes in the cellular redox potential (Price et al., 1994) or by detecting the products of ROS-inflicted damage (Evans et al., 2005). In addition, ROS can generate specific signaling effects through the peptides produced from proteolytic breakdown of oxidatively damaged proteins which act as secondary ROS messengers and contribute to a retrograde ROS signaling during different environmental challenges that generate oxidative stress (Moller and Sweetlove, 2010).

### Components Involved in ROS Signaling

**FIGURE 5:**
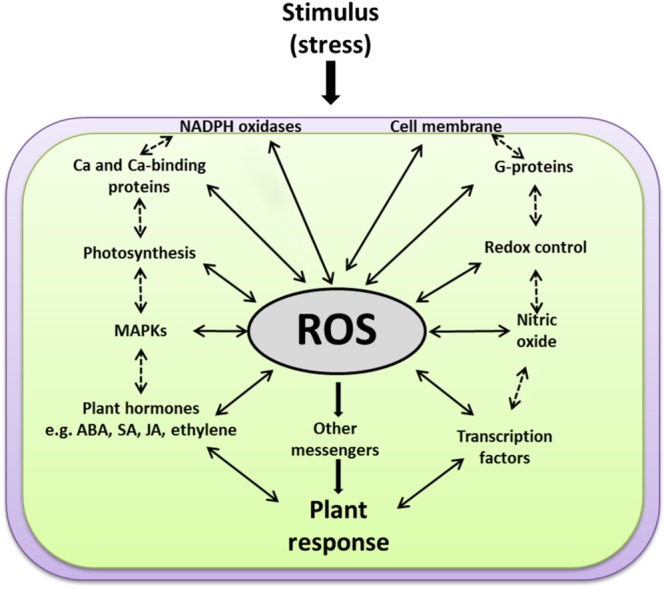
**A scheme explaining how ROS function at the cross-road of various key signaling events.** ROS work upstream and downstream of the other signaling components, e.g., membranes, NADPH oxidases, G-proteins, calcium, redox homeostasis, photosynthesis, MAPKs, plant hormones [such as salicylic acid (SA), jasmonic acid (JA), abscisic acid (ABA), and ethylene] and transcription factors. Solid arrows for direct ROS interactions with other signaling components, dashed arrows for expected indirect interactions.

The perceived ROS signals work upstream as well as downstream from many other second messengers in addition to many feedback and feedforward regulations in an interwoven manner to establish specific responses to different developmental and environmental cues. Currently, a major gap exists in our understanding of how ROS induce large-scale and coordinated expression from many genes. In addition, the big challenge is to identify the upstream sensing and signaling events through which ROS are perceived and delivered to the ROS-induced TFs. Do ROS activate the expression of TFs directly or through another set of signaling intermediates? How could secondary messengers such as G proteins, MAPKs, Ca^2+^, JA, SA, and ABA mediate the ROS signals and which one is upstream or downstream from each other? Does ROS, produced passively during different stresses through their damaging effects on cellular structures, induce signaling events that differ from those signaling events produced actively through activation of cell membrane-bound enzymes, like NADPH oxidases? The discussion below is an overview of the interconnectivity between ROS and other individual components involved in plant signal transduction pathways (**Figure 5**).

### Photosynthetic Activity

Photosynthesis represents the most peculiar feature that distinguishes plant and animal systems. In photosynthesis, through intersystem electron transport, the light energy captured by photosynthetic pigments is transformed into chemical energy which pumps reductants (NADPH) and ATP into the Calvin cycle (dark reactions) supplying carbon skeletons (sugars) for all major metabolic processes (Kromer, 1995). In addition to this role, plastids synthesize and store a large number of biomolecules, including carbohydrates, amino acids, fatty acids, and plant hormones (Buchanan et al., 2000). Therefore, it is self-evident that any change or imbalance in the function of the chloroplast will affect directly or/and indirectly the other cellular functions in plant cells. Consequently, environmental challenges can be primarily sensed via production of ROS and the concomitant changes of redox homeostasis of the chloroplast that act synergistically with other signaling pathways inducing then adaptive molecular and physiological responses (Huner et al., 1996). Earlier studies have suggested that the redox state of plastoquinone controls the rate of transcription of the chloroplast genes encoding reaction-center apoproteins of photosystem I and photosystem II (Pfannschmidt et al., 1999, 2009). Recently, it was reported that chloroplasts are able to sense light conditions and generate a remote control to modulate the nuclear gene expression to face the changing environment (Koussevitzky et al., 2007; Pogson et al., 2008; Pfannschmidt et al., 2009; Godoy Herz et al., 2014). As a retrograde signaling pathway, the redox state of components of the photosynthetic electron transport chain can sense the changes in photosysnthetic activity and in turn affect the nuclear gene expression (Fey et al., 2005; Nott et al., 2006; Godoy Herz et al., 2014). The plastidial metabolite, methylerythritol cyclodiphosphate (MEcPP), was found to regulate the expression of nuclear stress-response genes through a retrograde signal from the chloroplast to the nucleus (Xiao et al., 2012).

The thylakoid membranes and the involved redox complexes of the photosynthetic apparatus, especially the light energy harvesting PSII, are very sensitive targets to various environmental stress factors. During stress, excess of photosynthetically active light leads to the formation of ROS (e.g., ^1^O_2_ and OH^∙^; Glatz et al., 1999), which can induce membrane damage by attacking double bonds of unsaturated fatty acids. These effects are also expected to feedback signals for stress gene expression via the pathway which senses the physical state of the membrane (Glatz et al., 1999). This observation may reinforce the idea that receptors/sensors at the cell surface or the cell membranes could perceive stimuli, not directly from the stress, but indirectly through the chloroplast stress signals. It was reported that H_2_O_2_ from chloroplasts led to the induced expression of many genes coding for membrane-bound receptor proteins and signaling components (Sewelam et al., 2014a). In ozone-treated *Arabidopsis* leaves, Joo et al. (2005) reported that the chloroplastic ROS signal contributes to activating the membrane associated NADPH oxidases in intercellular signaling during the early component of the oxidative burst. Accordingly, they suggested that signaling from the chloroplast is central for oxidative stress induction by O_3_. Other studies also found that induction of light and stress response requires chloroplast signaling mediated by ROS (Allen et al., 1999; Fryer et al., 2003; Agrawal et al., 2004; Serrato et al., 2013).

Relatively little attention has been given to the role of photoproduced H_2_O_2_ and other ROS in defense signaling (Delledonne et al., 2001; Parker et al., 2001). This may be because the current models of signaling pathways controlling plant defense against pathogen infection are based mainly on animal models. Recently, new research has led to the development of models incorporating how the signaling pathways that are involved in light perception and in defense could operate and interact to form a complete defense signaling network, which includes systems to sense light and regulate gene expression. In this context, it was suggested that signals from the chloroplast and LESION SIMULATING DISEASE1 are integrated to mediate crosstalk between light acclimation and disease resistance in *Arabidopsis* (Mühlenbock et al., 2008). ROS produced from chloroplasts during the infection with *Xanthomonas campestris* play a major role in localized cell death in the non-host interaction between tobacco and this bacterial species (Zurbriggen et al., 2009). It was suggested that the chloroplast protein RPH1, a positive regulator of *Phytophthora brassicae*-induced oxidative burst, plays a role in the defense response of *Arabidopsis* and potato to *P. brassicae* (Belhaj et al., 2009). Based on these observations, it is clear that thylakoid membranes and hence photosynthesis, play a vital and very early role in stress sensing and signaling in plants, an idea that should be considered when constructing plant signaling networks.

### Redox Homeostasis

In plants, the continuous energy conversions, in the chloroplast and mitochondria, and the optimal use of the available light energy are only guaranteed when all reduction–oxidation (redox) processes are under precise control. Information on the redox situation is generated and transferred by various redox components, including various ROS and different antioxidants, that are parts of a robust network that links metabolism with regulation and signaling. Under environmental challenges, the imbalance in the network is sensed, and transformed into redox signals that are transmitted in order to elicit specific responses at various levels of regulation and in different subcellular compartments (Scheibe and Dietz, 2012). Thus, ROS and redox cues, generated under stress conditions, are essential to control the main metabolic processes through which cells convert and distribute the energy and metabolic fluxes, optimize different cell functions, activate acclimation responses through retrograde signaling, and control whole-plant systemic signaling pathways (Noctor, 2006; Suzuki et al., 2012). Redox homeostasis in the plant cell is considered to be an “integrator” of information from the environment controlling plant growth and stress responses, as well as cell death events (Dietz, 2003; Foyer and Noctor, 2009; Potters et al., 2010). The antioxidants, ascorbate, glutathione, carotenoids, and tocopherol, are information-rich redox buffers that affect numerous cellular components. In addition to their vital roles in stress response and as enzyme cofactors, cellular redox components influence plant growth and development by orchestrating processes from cell division to senescence and cell death (de Pinto and De Gara, 2004; Potters et al., 2004; Tokunaga et al., 2005; Halliwell, 2006). Most importantly, antioxidants influence gene expression associated with responses to different environmental cues to maximize defense through tuning cellular ROS levels and redox state (Foyer and Noctor, 2005). Proteins with oxidisable thiols such as glutathione and thioredoxin-1 are crucial for many functions of cell nuclei, including transcription, chromatin stability, nucleo-cytoplasmic trafficking, and DNA replication and repair (Nose, 2005; Go and Jones, 2010; Lukosz et al., 2010). From bacteria to humans, the triplet peptide, glutathione, is involved in protein S-glutathionylation where it regulates a variety of cellular processes by modulating protein function and prevents irreversible oxidation of protein thiols under unfavorable conditions (Dalle-Donne et al., 2009).

As redox homeostasis is greatly influenced by most, if not all, conditions that affect plant growth and development, the changes of intracellular redox determine various signaling events through their interaction with many other secondary messengers, such as protein kinases and phosphatases, phytohormones and calcium. Intensive current research might confirm that ROS-antioxidant interactions act as a metabolic interface between environmental changes and the concomitant signaling responses (Foyer and Noctor, 2005, 2012). For example, the redox state determined by the ROS-antioxidant interactions could regulate, directly or indirectly, the work of TFs, such as TGA, Athb-9, and RAP2 and hence the regulation of the expression of their downstream genes (Dietz, 2008).

### Membranes

The plasma membrane, as the selective barrier between living cells and their environments, plays a pivotal role in the perception of the changes in the surrounding environment (Guo et al., 2002). As a consequence of their rapid ability to modify their physical state, cellular membranes are not only the primary sites of stress damage, but also able to perceive environmental insults and activate remotely stress-defense genes (Glatz et al., 1999). The microdomain organization and physical state of cell membranes is known to be a very sensitive monitor of different environmental challenges (Horvath et al., 1998). It was stated that heat stress changes the membrane fluidity and H_2_O_2_ responds rapidly to this change, leading to the activation of small heat shock protein synthesis (Königshofer et al., 2008).

As a component of cell membranes, ion channels play a vital role in the transduction of environmental and internal signals (Binder et al., 2003). Ion channels are proteins forming hydrophilic pathways through the plasma membranes (Barnes et al., 1997). They function as permeation pores through which the electrically charged species can pass across biological membranes. It was reported that ion channels are directly involved in important cellular processes, such as plant defense responses induced by elicitors (Czempinski et al., 1997), light perception (Ermolayeva et al., 1997), and mechanical signals (Cosgrove and Hedrich, 1991). For example, it was reported that the efficiency of H_2_O_2_ signaling between cells is controlled by plasma membrane aquaporin pores where the expression of several plant plasma membrane aquaporins in yeast, such as *Arabidopsis* plasma membrane intrinsic protein PIP2.1, was found to enhance the toxicity of H_2_O_2_ when yeast cells were exposed to H_2_O_2_ treatment (Dynowski et al., 2008). It was found that the disruption of a cyclic nucleotide-gated calcium channel gene causes a hyper-thermosensitive phenotype in *Arabidopsis* and moss indicating that the plasma membrane cyclic nucleotide-gated calcium channels control plant thermal sensing and acquired thermotolerance (Saidi et al., 2009; Finka et al., 2012).

During exposure to stress, the major role of phospholipids, the backbone of cellular membranes, may be to serve as precursors for the generation of secondary messenger signaling molecules, such as phosphotidylinositol, inositol 1,4,5-tiphosphate (IP3), diacylglycerol, and jasmonates. IP3 and diacylglycerol are secondary messengers that can activate protein kinase and induce Ca^2+^ release, respectively. Additionally, IP3 itself is a signal and may be involved in several processes, such as the recruitment of signaling complexes to specific membrane location and their assembly (Guo et al., 2002). In addition, under oxidative stress, polyunsaturated fatty acids (PUFAs) are attacked by different ROS, specially ^1^O_2_ and OH^∙^. This causes production of lipid hydroperoxides, leading to a decrease of membrane fluidity (Møller et al., 2007). In turn, this change in membrane physical state could activate downstream signaling intermediates.

### NADPH Oxidases

Membrane-bound NADPH oxidases are a group of enzymes that catalyze the production of superoxide radicals ($O_{2}^{•-}$) in animals and plants (Sagi and Fluhr, 2006). In mammals, NADPH oxidases are also called respiratory burst oxidases (Rbo). Because of their functional homology with mammals, plant NADPH oxidases are known as respiratory burst oxidase homolog (Rboh; Torres and Dangl, 2005). In plants, Rboh enzymes are the source of ROS production under pathogen infection and in many of other processes (Torres and Dangl, 2005). The ability of Rboh enzymes to integrate various signaling players, such as calcium and protein phosphorylation with ROS production, suggests a crucial role for Rboh in many different biological processes in cells, and places them at the core of the ROS signaling network of cells, revealing their important functions in plants (Suzuki et al., 2012; Kadota et al., 2014).

In *Arabidopsis*, there are ten *Rboh* genes (Torres et al., 1998; Dangl and Jones, 2001). Many studies have reported the induction of *Rboh* gene expression by pathogens and fungal elicitors (Simon-Plas et al., 2002; Yoshioka et al., 2003; Wang et al., 2006). In addition, using mutant analysis, it was suggested that RbohD and RbohF are required for ROS production and cell death in *Arabidopsis* plants infected with *P. syringae* or *Peronospora parasitica* (Torres et al., 2002). The same group (Torres et al., 2005) reported that RbohD is required for ROS production but this ROS antagonizes cell death induced by *Pseudomonas* infection. In *Nicotiana benthamiana*, silencing of *NbrbohA* and *NbrbohB* led to reduction of ROS production and reduced resistance to *Phytophthora infestans* infection (Yoshioka et al., 2003). The *Arabidopsis* RbohF was suggested to be a vital player in defense-associated metabolism and a key factor in the interaction between oxidative stress and pathogen infection (Chaouch et al., 2012). Regarding the involvement of Rboh in abiotic interactions, it was reported that the *Arabidopsis RbohD* gene is involved in ROS-inducing a rapid systemic signal during various stress factors, such as heat, cold, high light, and salinity (Miller et al., 2009). The abiotic stress-mediating phytohormone ABA was reported to be regulated by the action of RbohD and RbohF in different ROS-ABA signaling pathways (Kwak et al., 2003; Joo et al., 2005; Xue and Seifert, 2015). In a microarray experiment, *RbohD* expression was downregulated by ABA treatment but upregulated by H_2_O_2_ treatment in *Arabidopsis* (Barraud et al., 2006). During salt stress, ROS produced by both AtrbohD and AtrbohF seem to function as signal molecules to regulate Na^+^/K^+^ homeostasis, where the two *Arabidopsis* double mutants *atrbohD1*/*F1* and *atrbohD2*/*F2* were found to produce less ROS and to be much more sensitive to NaCl treatments than wild-type (Ma et al., 2012). RbohD was found to contribute to the ROS-responsive expression of *ERF6*, a ROS regulator TF in *Arabidopsis* (Sewelam et al., 2013). As NADPH oxidases are physically located at the plasma membrane, they are proposed to play an early and vital signaling role and should be highly considered when constructing plant signaling networks.

### G Proteins

GTP-binding proteins (G proteins) are found in almost all organisms from prokaryotes to humans (Assmann, 2004). G proteins mediate stimulus perception by G-protein-coupled receptors (GPCR), in addition to other regulatory proteins. In humans, there are about 1000 GPCR, representing the largest group of cell surface receptors encoded by mammalian genome (Nagarathnam et al., 2012). It is estimated that about 60% of all drugs currently available target G-protein-based pathways and G protein component disorders have been found to cause various genetic diseases (Assmann, 2004). G proteins are heterotrimeric proteins composed of three monomers; α, β, and γ. About 20 G protein α subunits (Gα), 6 Gβ subunits, and 20 Gγ subunits have been characterized in mammals (Gutkind, 2000). Controversially, in plants the situation seems to be much simpler than that in animal systems. For example, it was reported that the *Arabidopsis* genome encodes only single Gα and Gβ subunits, two Gγ subunits, just one GPCR protein, and one regulator of G protein signaling (Assmann, 2004).

The involvement of G proteins in plant stress signaling is evident, especially in plant-pathogen interactions (Assmann, 2005; Trusov et al., 2009; Maruta et al., 2015; Xu et al., 2015). Regarding ROS, many studies have suggested a tight relationship between ROS and G proteins in stress-mediated plant signaling. It was reported that, on exposure of *Arabidopsis* leaves to ozone, the first biphasic oxidative burst is greatly attenuated or completely absent in mutant plants lacking Gα protein or Gβ protein. This finding suggests that the ROS produced by ozone in the apoplastic fluid do not themselves enter cells to activate intracellular ROS-producing systems. Rather, the extracellular ROS activate the G protein either directly or indirectly (Joo et al., 2005). It is possible that G proteins themselves are directly activated by ROS. In this regard, it has been reported that two mammalian Gα proteins, Gαi and Gαo, are redox-controlled (Fujiki et al., 2000). The membrane-bound NADPH oxidases D and F were suggested to receive initial signals from G proteins to mediate ozone responses in *Arabidopsis* guard cells (Suharsono et al., 2002). The absence of the Gα subunit in the *gpa1* mutant disrupts the interplay between ABA perception and ROS production, with a consequent inhibition of Ca^2+^-channel activation (Zhang et al., 2011). The membrane-bound ROS producing enzymes AtRbohD and AtRbohF work in the same pathway with the Gβ subunit of the heterotrimeric G protein for full disease resistance to different *P. syringae* strains (Torres et al., 2013). In plants, further studies are required to unravel the roles of G proteins and their signaling roles.

### Calcium Signaling

The use of calcium ions as a secondary messenger represents an integral part in many signal transduction pathways in all life forms, from vertebrate animals to plants (Berridge et al., 2000; Stael et al., 2012). In contrast to other similar ions, such as Mn^2+^, the Ca^2+^ ion has many peculiar features, including a favorable ionic radius and hydration status, an irregular geometry, and flexible coordination chemistry (Jaiswal, 2001). The main calcium stores in plant cells are: the vacuole, the endoplasmic reticulum and the apoplast (Stael et al., 2012). Elevation in cytoplasmic Ca^2+^ represents an early response to many different biotic and abiotic stresses, including oxidative (McAinsh and Pittman, 2009; Dodd et al., 2010). As a second messenger in a wide range of signaling pathways in plants, calcium connects the perception of different stimuli and stresses to their downstream cellular responses (Evans et al., 2005). It has been stated that transient cellular calcium elevations are sensed by several Ca^2+^ sensors such as calmodulin (CAM), calmodulin-like (CML), calcium-dependent protein kinase (CDPK), and calcineurin B-like protein (CBL; Bouche et al., 2005; McCormack et al., 2005; Das and Pandey, 2010; Asai et al., 2013). A direct interconnection between CBL-CIPK-mediated Ca^2+^ and ROS signaling in plants was reported as evidence for a synergistic activation of the NADPH oxidase RbohF by direct Ca^2+^-binding to its EF-hands (Drerup et al., 2013). The *Arabidopsis* CPK5, an isoform of the plant CDPK family, was activated rapidly in response to infection with *P. syringae*, resulting in Rboh-mediated ROS production and enhanced SA-mediated resistance to this bacterial pathogen (Dubiella et al., 2013). In the same study, RbohD was reported to be an *in vivo* phosphorylation target of CPK5. Ca^2+^ ions also regulate long-distance root-to-shoot signaling and may also have roles in transmitting ROS signals (Choi et al., 2014).

The concentration of cytosolic Ca^2+^, the expression level of calmodulin 1 (*CAM1*) gene, the content of CAM proteins and the expression of many antioxidant genes in maize are increased after treatment with ABA or H_2_O_2_. Furthermore, pre-treating plants with CAM inhibitors almost completely blocked the upregulation of many antioxidant enzymes (Hu et al., 2007). These findings show that the increase in cytosolic Ca^2+^ requires CAM to deliver its signal to the downstream targets. Ca^2+^ elevations have been suggested, in some cases, to be upstream of ROS production; in other cases, Ca^2+^ elevations have been reported to be downstream of ROS production (Bowler and Fluhr, 2000). Several workers showed that oxidative stress results in increased cytosolic Ca^2+^. In tobacco seedlings, oxidative stress stimulates cytosolic Ca^2+^ increases (Bhattacharjee, 2005). The allelopathic toxin catechin was reported to cause rapid ROS production, followed by ROS-induced Ca^2+^ increases in diffuse knapweed (*Centaurea diffusa*) and *Arabidopsis* roots (Bais et al., 2003). It was reported that pre-treatment of *Arabidopsis* plants with the calcium channel blocker lanthanum chloride (LaCl_3_) attenuated the inducing effect of H_2_O_2_ on *ERF6*, suggesting that Ca^2+^ is playing a signaling role, which is downstream from ROS, in the induction of this TF by H_2_O_2_ (Sewelam et al., 2013). On the contrary, other research groups have reported that Ca^2+^ works upstream of ROS. For example, it was reported that inhibitors of Ca^2+^ fluxes inhibit both increase in cytosolic Ca^2+^ and H_2_O_2_, whereas inhibitors of NADPH oxidase blocks only the oxidative burst (Abuharbeid et al., 2004). Mechanical forces (e.g., touch) were found to trigger rapid and transient increases in cytosolic Ca^2+^ and to stimulate apoplastic ROS production. The production of ROS was inhibited by pre-treatment with Ca^2+^ channel blockers (Monshausen et al., 2009), suggesting a role for Ca^2+^ as a prerequisite of ROS production under mechanical stimuli. To avoid this ostensible contradiction, future studies should consider the presence of a large number of sources for ROS production as well as a plethora of Ca^2+^ subcellular sources, in addition to the expected feedback mechanisms. Nevertheless, these studies, at least, designate a crucial role for ROS-Ca^2+^ signaling during plant responses to stresses that should be considered when constructing global plant signaling networks.

### Nitric Oxide (NO)

Nitric oxide (NO) is a small, water-, and lipid-soluble free radical gas with well-characterized signaling roles in mammalian systems (Furchgott, 1995; Neill et al., 2003; Moreau et al., 2010). Nitric oxide production by plants and its involvement in plant growth were described in the late 1970s (Anderson and Mansfield, 1979; Klepper, 1979). Research on the effects of NO in plants focused on atmospheric pollution by the oxides of nitrogen, NO and NO_2_ (nitrogen dioxide; Hufton et al., 1996). It was revealed that plants not only respond to atmospheric NO, but also produce considerable amounts of endogenous NO (Wildt et al., 1997). However, research on NO as a signaling molecule in plants started with the work done by Leshem and Haramaty (1996) and became well-established after the description of its role in plant defense signaling (Delledonne et al., 1998; Durner et al., 1998, 1999). Currently, it is well-known that NO plays an important signaling role in plant growth, development and defense responses (Besson-Bard et al., 2008; Moreau et al., 2010). It was reported that ROS and NO are produced concomitantly under various stresses and can interact with each other to induce a defense response (Neill et al., 2002; Yoshioka et al., 2009; Molassiotis and Fotopoulos, 2011). NO could have toxic or protective effects, depending on its concentration, combination with ROS compounds, and its subcellular localization (Correa-Aragunde et al., 2015).

Many reports have suggested interconnected signaling roles between ROS and NO during plant response to different stresses. Generation of NO at the same time as H_2_O_2_ in response to pathogen infection was found to mediate defense responses similar to those seen following H_2_O_2_ production (Delledonne et al., 1998; Durner et al., 1998; Asai and Yoshioka, 2009; Del Río, 2015). Delledonne et al. (1998) reported that treatment of soybean cultures with avirulent *P. syringae* induces rapid NO synthesis with kinetics similar to H_2_O_2_ generation, indicating an interaction between NO and H_2_O_2_ during plant response to pathogen attack. NO biosynthesis was reported to be regulated by H_2_O_2_-mediated activation of MAP Kinase 6 in *Arabidopsis* (Wang et al., 2010). A proteomic study on salt-stressed citrus plants pre-treated with H_2_O_2_ or NO has suggested an overlap between H_2_O_2_ and NO signaling pathways in acclimation to salinity (Tanou et al., 2009, 2010). Under drought stress, it was suggested that ROS and NO interact to induce ABA biosynthesis to affect stomatal closure (reviewed by Neill et al., 2003). Regarding the mechanisms by which NO exerts its effects, it is suggested that NO may deliver its signaling roles via modulating the activity of proteins through nitrosylation and probably tyrosine nitration, in addition to the role that NO can act as a Ca-mobilizing messenger (Besson-Bard et al., 2008). ABA signaling in guard cells was found to be negatively regulated by NO through *S*-nitrosylation-mediated inhibition of the open stomata 1 (OST1)/sucrose non-fermenting 1 (SNF1)-related protein kinase 2.6 (SnRK2.6; Wang et al., 2015). In fact, NO can interact with ROS in different ways and might work as an antioxidant molecule during various stresses (Beligni and Lamattina, 1999; Correa-Aragunde et al., 2015). Moreover, modulation of superoxide formation by NO (Caro and Puntarulo, 1998) and inhibition of lipid peroxidation (Boveris et al., 2000) could illustrate a potential antioxidant role for NO. The oxidative damage in sorghum embryos was found to be alleviated by pre-treatment with sodium nitroprusside and diethylenetriamine NONOate as sources of exogenous NO (Jasid et al., 2008). Alternatively, excess NO can result in nitrosative stress (Hausladen et al., 1998), so a positive balance of ROS/NO is essential.

### Mitogen-Activated Protein Kinases

Mitogen (induces mitotic division)-activated protein kinases (MAPKs) are evolutionary conserved enzymes. In eukaryotes, signaling pathways arbitrated by MAPKs have been considered as a general signal transduction mechanism that links different receptors to their cellular and nuclear targets (Tena et al., 2001). The signaling events mediated by MAPKs are composed of three functionally interlinked protein kinases: a MAP kinase kinase kinase (MAPKKK), a MAP kinase kinase (MAPKK), and a MAP kinase (MAPK; Rodriguez et al., 2010; Sinha et al., 2011). In this phosphorylation module, a MAPKKK phosphorylates and activates a particular MAPKK, which in turn phosphorylates and activates a MAPK by phosphorylation of the tyrosine and therionine residues in the TXY motif (Qi and Elion, 2005).

In the *Arabidopsis* genome, 20 MAPK, 10 MAPKK, and 60 MAPKKK encoding genes were identified (Ichimura et al., 2002). The current functional analysis of MAPK cascades, mainly in *Arabidopsis*, revealed that plants have an overall of 24 MAPK pathways of which only a small set has been investigated so far (Wrzaczek and Hirt, 2001). This may reflect why MAPK signaling cascades are so complicated. The challenge ahead is to describe the elements of plant MAPK cascades and to specify roles of individual MAPK cascade genes, in particular signaling pathways (Wrzaczek and Hirt, 2001). The spatial and temporal expression and interaction characteristics of MAPKs are suggested to define their specificity in different signaling pathways (Dietz et al., 2010). The *Arabidopsis* mitogen-activated protein kinase 8 (MPK8) was reported to connect protein phosphorylation, Ca^2+^, and ROS in wound signaling pathways (Takahashi et al., 2011).

Some of the components of MAPK cascades are elicited by cold, drought, H_2_O_2_, heat, wounding, pathogens, elicitors, ABA, SA, and ethylene (reviewed by Bowler and Fluhr, 2000). In many eukaryotes, the transmission of oxidative signals is controlled by protein phosphorylation involving MAPKs (Kyriakis and Avruch, 1996; Gustin et al., 1998; Pitzschke and Hirt, 2006; Xing et al., 2008). On the one hand, MAPKs can be activated by accumulation of H_2_O_2_, on the other hand they can trigger an H_2_O_2_-induced oxidative burst (Nakagami et al., 2005; Pitzschke et al., 2009; reviewed by Petrov and Van Breusegem, 2012). In *Nicotiana benthamiana*, the MAPK cascades MEK2-SIPK/NTF4 and MEK1-NTF6 were reported to participate in the regulation of the radical burst induced by the oomycete pathogen *P. infestans* through NO and RbohB-dependant ROS generation (Asai et al., 2008). Using *Arabidopsis* protoplasts, a correlation was revealed between the activation of plant MAPK cascade and H_2_O_2_, which is generated by various stress factors. In this study, it was observed that H_2_O_2_ activates the MAPKKK, ANP1, which in turn phosphorylates the downstream kinases, AtMPK3 and AtMPK6 (Kovtun et al., 2000). Protein phosphorylation through MAPK cascades was suggested to trigger a positive feedback regulation of Ca^2+^ and ROS via the activation of RbohD and RbohF in *Arabidopsis* (Kimura et al., 2012). A maize MAPK, MAP65-1a, was reported to positively control H_2_O_2_ amplification and to enhance the antioxidant enzymes SOD and APX through the brassinosteroid signaling pathway (Zhu et al., 2013). The expression of the *Arabidopsis OXI1* gene, encoding a serine/threonine kinase, was induced in response to a broad range of H_2_O_2_-producing stimuli and OXI1 kinase activity itself was also induced by H_2_O_2_ *in vivo* (Rentel et al., 2004). Application of bioinformatics and computational analysis would be required to illuminate how different MAPKs coordinate different plant signaling events.

### Abscisic Acid (ABA)

Substantial evidence postulates that ABA plays a vital role in controlling downstream responses essential for adaptation to stress (Leung and Giraudat, 1998; Raghavendra et al., 2010). These responses include changes in stomatal conductance, growth, osmolyte accumulation, and gene expression (Chen et al., 2002; Verslues and Zhu, 2004; Krasensky and Jonak, 2012). In contrast to the positive role of ABA in abiotic stress response, ABA has been considered as a negative regulator of disease resistance. This negative effect appears to be due to the obstruction by ABA of biotic stress signaling pathways that are orchestrated by SA, JA, and ethylene (Coego et al., 2005). ABA can also improve disease resistance by modifying cell wall deposits, such as callose (Mauch-Mani and Mauch, 2005).

A simultaneous enhanced level of ROS and ABA in plant tissues has been monitored under different types of environmental stresses. The concomitant enhancement of ROS and ABA during stress has been suggested to be a node in cross-tolerance to multiple types of stresses (Verslues and Zhu, 2004). It has been indicated that ROS generated by NADPH oxidases work downstream of ABA in mediating stomatal closure during stress (reviewed by Verslues and Zhu, 2004). It was reported that the production of H_2_O_2_ in the chloroplasts, mitochondria and peroxisomes under water stress was abolished in the leaves of maize plants pre-treated with the ABA biosynthesis inhibitor (tungstate) or in an ABA mutant plants, indicating that ABA is required for H_2_O_2_ production in these compartments (Hu et al., 2006). It was demonstrated that a temporal-spatial interaction between ROS and ABA regulates rapid systemic acquired acclimation to environmental challenges in plants (Suzuki et al., 2013). In response to heat and oxidative stresses, it was reported that H_2_O_2_ mediates a crosstalk between the plant hormones; brassinosteroid and ABA, via a signaling pathway through which brassinosteroid induces a rapid and transient H_2_O_2_ production by NADPH oxidase. The process in turn activates increased ABA levels, leading to further increases in H_2_O_2_ production and improved stress tolerance in tomato plants (Zhou et al., 2014).

### Salicylic Acid, Jasmonic Acid, and Ethylene

Various plant developmental and stress responses require a tuned coordination between the phytohormones SA, JA and ethylene. It is thought that, in *Arabidopsis*, a JA-ethylene signaling pathway is important to mediate resistance to necrotrophic pathogens (feed on dead tissues), such as *Botrytis cinerea*. On the other hand, the SA signaling pathway is supposed to mediate resistance to biotrophic pathogens (feed on living tissues), such as *P. syringae* (Thomma et al., 2001; Anderson et al., 2004). However, it has been suggested that many genes are co-regulated by these hormones and there is considerable genetic evidence for crosstalk between these signaling pathways (Schenk et al., 2000; Glazebrook et al., 2003; Leon-Reyes et al., 2009). Regarding ROS signaling, it was suggested that SA, JA, and ethylene work together with ROS and play crucial regulatory roles in plant defense responses (Mur et al., 2006; Mhamdi et al., 2010).

**Salicylic acid (SA)** is well-known to regulate both systemic acquired resistance (SAR) and local disease resistance mechanisms, including host cell death and defense gene expression (Park et al., 2007; Vlot et al., 2008). It was reported that SA elicits an oxidative burst, which in turn promotes SAR (Senaratna et al., 2000). One of the proposed modes of action of SA is the inhibition of catalase, a major enzyme scavenging H_2_O_2_, thereby increasing cellular concentrations of H_2_O_2_, which acts as a second messenger and activates defense-related genes (Ananieva et al., 2002). The extracellular production of ROS was found to enhance SA production inducing stomatal closure in *Arabidopsis* (Khokon et al., 2011). It was reported that SA accumulation in *siz1* mutant plants enhances stomatal closure and drought tolerance through controlling ROS accumulation in *Arabidopsis* guard cells (Miura et al., 2013). It was suggested that SA accumulation and signaling is activated by increased H_2_O_2_ levels through changes in the glutathione pool in an *Arabidopsis* catalase 2 (cat2) mutant (Han et al., 2013a).

**Jasmonic acid (JA)** and methyl jasmonate (MeJA), are natural products regulating plant development, response to environmental challenges, and gene expression (Bell et al., 1995). A signaling role for JA in defense responses has been suggested in plants (Farmer and Ryan, 1992; Wasternack and Hause, 2013). Currently, there is accumulating evidence suggesting a strong relationship between ROS and JA signaling. For instance, it was suggested that MeJA pretreatment of *Arabidopsis* inhibited O_3_-induced H_2_O_2_ production and SA accumulation and completely abolished O_3_ induced cell death (Rao et al., 2000). It was reported that ROS generated by RbohD and RbohF enzymes are important for JA-induced expression of genes regulated by MYC2, a TF involved in the JA-mediated response, where treating *RbohD* and *RbohF* mutant plants with MeJA failed to increase the expression levels of various MYC2 downstream genes (Maruta et al., 2011). A dynamic interaction between JA and ROS was characterized to regulate lignin biosynthesis in response to cell wall damage where ROS produced by RbohD and JA-isoleucine generated by JASMONIC ACID RESISTANT1 were found to form a negative feedback loop that influence lignin accumulation (Denness et al., 2011). It was revealed that the intracellular ROS production in *cat2* mutant *Arabidopsis* plants leads to activating the JA pathway and its related genes with glutathione accumulation as an intermediate (Han et al., 2013b).

**Ethylene (C_2_H_4_)** is one of the simplest organic molecules that have biological activity in plants (Zarembinski and Theologis, 1994). It is well-documented that ethylene is a main player in PCD, either during senescence (Orzaez and Granell, 1997), oxidative stress imposed by ozone (Overmyer et al., 2000), or plant pathogen interactions (Lund et al., 1998). In addition, it was suggested that ethylene is crucial for H_2_O_2_ production during PCD in tomato suspension cells (de Jong et al., 2002). It was reported that the ethylene receptor ETR1 mediates H_2_O_2_ signaling in guard cells in *Arabidopsis* (Desikan et al., 2005). Together, these findings suggest a cross-talk between ethylene and ROS in plant signaling. Treating *Arabidopsis* plants with the bacterial elicitor flagellin (flg22) enhanced an oxidative burst which was inhibited in ethylene-insensitive mutants, *etr1* and *ein2*, indicating a requirement of ethylene signaling for ROS production (Mersmann et al., 2010). A synergistic biosynthesis of ethylene and ROS production, mediated by the plasma membrane bound enzymes RbohD and RbohF, was reported in tobacco plants infected with the hemibiotrophic *Phytophthora parasitica* (Wi et al., 2012).

### Transcription Factors

Regulation of gene expression at the transcriptional level influences or controls many of the biological processes in a cell or organism, such as progression through the cell cycle, metabolic and physiological balance, and responses to environment (Riechmann et al., 2000). Plant stress responses are regulated by multiple signaling pathways that activate gene transcription and its downstream machinery. Using data from ROS-related microarray studies, Gadjev et al. (2006) examined the expression of 1,500 TFs in *Arabidopsis* in response to different ROS, including singlet oxygen, H_2_O_2_ and OH^∙^. They reported that different ROS induced or repressed the expression of about 500 of these annotated TFs in *Arabidopsis*. Nevertheless, the transcriptional regulation mechanisms mediating ROS signaling is not fully understood. It is suggested that the regulation of the TFs activity by the most important ROS, H_2_O_2_, is managed at several levels including: (1) upregulation of TF expression or increasing both mRNA stability and translation; (2) increasing the stability of the TF by decreasing its association with the protein degrading ubiquitin E3 ligase complex or by inhibiting this complex; (3) nucleo-cytoplasmic traffic by transferring or masking nuclear localization signals, or by releasing the TF from partners or from membrane anchors; and (4) DNA binding and nuclear transactivation by adapting TF affinity toward DNA, co-activators or repressors, and by targeting specific regions of chromatin to activate individual genes (Marinho et al., 2014). Many examples of TFs that are regulated by ROS have been revealed. Simple organisms, such as bacteria and yeast, sense the enhanced production of ROS using redox sensitive TFs (Mittler et al., 2004). In bacteria, OxyR (oxygen regulated) and PerR (Peroxide Regulon Repressor) are TFs that are directly activated by H_2_O_2_. The tetrameric OxyR protein is characterized as a regulatory protein that activates nine out of twelve early H_2_O_2_-induced proteins. The OxyR transcription activator exists in two forms, reduced and oxidized; only the oxidized state is able to initiate transcription (Storz et al., 1990; Storz and Imlayt, 1999). In yeast, four TFs, namely, Yap1, Maf1, Hsf1, and Msn2/4, were reported to be regulated by ROS. For example, Yap1 is regulated by H_2_O_2_ at the level of nucleo-cytoplasmic trafficking. Under oxidative stress, the export of Yap1 to the nucleus is decreased and Yap1 is kept longer in the nucleus where it regulates its target genes (Delaunay et al., 2000). Yap1 has a key role in the oxidative stress response, redox homeostasis and electrophilic response, regulating the transcription of genes encoding antioxidant and detoxification enzymes in yeast cells (Marinho et al., 2014). In multicellular organisms, nine different TFs, namely AP-1, NRF2, CREB, HSF1, HIF-1, TP53, NF- κB, NOTCH, SP1, and SCREB-1, are well-characterized to be regulated by ROS (Marinho et al., 2014).

## Conclusion

As reviewed here, plants have evolved complicated protection mechanisms to survive different environmental challenges. The recent functional molecular and physiological studies have produced new details attempting to unravel the complexity of these signaling pathways. It is evident now that there is no a specific linear signaling pathway for each stress, instead, there are interconnected networks including common signaling events that are shared by various pathways represented by what we call cross-talk. From these big signaling networks many branches arise for specificity. As ROS are well-known to be produced by plants in response to different biotic and abiotic stresses, they are designated to work at the cross road within these complex signaling networks. ROS play this central signaling role through their evident interactions, whether upstream or downstream, with other key signaling components, including membranes, NADPH oxidases, G-proteins, calcium, redox homeostasis, MAPKs, plant hormones (such as SA, JA, ABA, and ethylene) and TFs. The recent research implies an early and vital role for photosynthesis in sensing various environmental insults, not only abiotic, but also biotic ones, a concept that needs to be taken into consideration when studying stress signaling pathways in plants. Despite all of these achievements, great efforts are still required to be able to reconstruct larger signaling networks that may include ROS at the convergent points. In this regard, bioinformatics and systems biology approaches are nominated to greatly help in constructing global signaling networks. As a result, these global networks would improve our understanding of plant biology and assist us to develop new strategies for higher plant productivity in the face of increasingly severe environmental conditions and the high demand for food, fiber, and energy crops.

## Author Contributions

All authors wrote and approved the final version of the manuscript.

## Conflict of Interest Statement

The authors declare that the research was conducted in the absence of any commercial or financial relationships that could be construed as a potential conflict of interest.

## References

[B1] Abuharbeid S. Apel J. Sander M. Fiedler B. Langer M. Zuzarte M. L. (2004). Cytotoxicity of the novel anti-cancer drug rViscumin depends on HER-2 levels in SKOV-3 cells. *Biochem. Biophys. Res. Commun.* 321 403–412. 10.1016/j.bbrc.2004.06.160 15358191

[B2] Agrawal V. Zhang C. Shapiro A. D. Dhurjati P. S. (2004). A dynamic mathematical model to clarify signaling circuitry underlying programmed cell death control in *Arabidopsis* disease resistance. *Biotechnol. Prog.* 20 426–442. 10.1021/bp034226s 15058987

[B3] Akter S. Huang J. Waszczak C. Jacques S. Gevaert K. Van Breusegem F. (2015). Cysteines under ROS attack in plants: a proteomics view. *J. Exp. Bot.* 66 2935–2944. 10.1093/jxb/erv044 25750420

[B4] Allen G. J. Kwak J. M. Chu S. P. Llopis J. Tsien R. Y. Harper J. F. (1999). Cameleon calcium indicator reports cytoplasmic calcium dynamics in *Arabidopsis* guard cells. *Plant J.* 19 735–747. 10.1046/j.1365-313x.1999.00574.x 10571859

[B5] Allen G. J. Muir S. R. Sanders D. (1995). Release of Ca^2+^ from individual plant vacuoles by both InsP3 and cyclic ADP-ribose. *Science* 268 735–737. 10.1126/science.7732384 7732384

[B6] Ananieva E. A. Alexieva V. S. Popova L. P. (2002). Treatment with salicylic acid decreases the effects of paraquat on photosynthesis. *J. Plant Physiol.* 159 685–693. 10.1078/0176-1617-0706

[B7] Anderson J. P. Badruzsaufari E. Schenk P. M. Manners J. M. Desmond O. J. Ehlert C. (2004). Antagonistic interaction between abscisic acid and jasmonate-ethylene signaling pathways modulates defense gene expression and disease resistance in *Arabidopsis*. *Plant Cell* 16 3460–3479. 10.1105/tpc.104.025833 15548743 PMC535886

[B8] Anderson L. S. Mansfield T. A. (1979). The effects of nitric oxide pollution on the growth of tomato. *Environ. Pollut.* 20 113–121. 10.1016/j.jplph.2010.04.007

[B9] Angers B. Castonguay E. Massicotte R. (2010). Environmentally induced phenotypes and DNA methylation: how to deal with unpredictable conditions until the next generation and after. *Mol. Ecol.* 19 1283–1295. 10.1111/j.1365-294X.2010.04580.x 20298470

[B10] Apel K. Hirt H. (2004). Reactive oxygen species: metabolism, oxidative stress, and signal transduction. *Annu. Rev. Plant Biol.* 55 373–399. 10.1146/annurev.arplant.55.031903.141701 15377225

[B11] Asada K. (1999). The water-water cycle in chloroplasts: scavenging of active oxygens and dissipation of excess photons. *Annu. Rev. Plant Physiol. Plant. Mol. Biol.* 50 601–639. 10.1146/annurev.arplant.50.1.601 15012221

[B12] Asada K. (2006). Production and scavenging of reactive oxygen species in chloroplasts and their functions. *Plant Physiol.* 141 391–396. 10.1104/pp.106.082040 16760493 PMC1475469

[B13] Asai S. Ichikawa T. Nomura H. Kobayashi M. Kamiyoshihara Y. Mori H. (2013). The variable domain of a plant calcium-dependent protein kinase (CDPK) confers subcellular localization and substrate recognition for NADPH oxidase. *J. Biol. Chem.* 288 14332–14340. 10.1074/jbc.M112.448910 23569203 PMC3656289

[B14] Asai S. Ohta K. Yoshioka H. (2008). MAPK signaling regulates nitric oxide and NADPH oxidase-dependent oxidative bursts in *Nicotiana benthamiana*. *Plant Cell* 20 1390–1406. 10.1105/tpc.107.055855 18515503 PMC2438462

[B15] Asai S. Yoshioka H. (2009). Nitric oxide as a partner of reactive oxygen species participates in disease resistance to nectrotophic pathogen *Botryis cinerea* in *Nicotiana benthamiana*. *Mol. Plant Microbe. Interact.* 22 619–629. 10.1094/MPMI-22-6-0619 19445587

[B16] Asai T. Stone J. M. Heard J. E. Kovtun Y. Yorgey P. Sheen J. (2000). Fumonisin B1-induced cell death in *arabidopsis* protoplasts requires jasmonate-, ethylene-, and salicylate-dependent signaling pathways. *Plant Cell* 12 1823–1836. 10.2307/3871195 11041879 PMC149122

[B17] Assmann S. M. (2004). Plant G proteins, phytohormones, and plasticity: three questions and a speculation. *Sci. STKE* 2004:re20. 10.1126/stke.2642004re20 15613689

[B18] Assmann S. M. (2005). G protein regulation of disease resistance during infection of rice with rice blast fungus. *Sci. STKE* 2005:cm13. 10.1126/stke.3102005cm13 16291770

[B19] Atkinson N. J. Urwin P. E. (2012). The interaction of plant biotic and abiotic stresses: from genes to the field. *J. Exp. Bot.* 63 3523–3543. 10.1093/jxb/ers100 22467407

[B20] Baena-González E. (2010). Energy signaling in the regulation of gene expression during stress. *Mol. Plant* 3 300–313. 10.1093/mp/ssp113 20080814

[B21] Baginsky S. Hennig L. Zimmermann P. Gruissem W. (2010). Gene expression analysis, proteomics, and network discovery. *Plant Physiol.* 152 402–410. 10.1104/pp.109.150433 20018595 PMC2815903

[B22] Bais H. P. Vepachedu R. Gilroy S. Callaway R. M. Vivanco J. M. (2003). Allelopathy and exotic plant invasion: from molecules and genes to species interactions. *Science* 301 1377–1380. 10.1126/science.1083245 12958360

[B23] Bardo S. Robertson B. Stephens G. J. (2002). Presynaptic internal Ca^2+^ stores contribute to inhibitory neurotransmitter release onto mouse cerebellar Purkinje cells. *Br. J. Pharmacol.* 137 529–537. 10.1038/sj.bjp.0704901 12359635 PMC1573523

[B24] Barnes S. A. McGrath R. B. Chua N. H. (1997). Light signal transduction in plants. *Trends Cell Biol.* 7 21–26. 10.1016/S0962-8924(97)10043-5 17708894

[B25] Barraud N. Hassett D. J. Hwang S. H. Rice S. A. Kjelleberg S. Webb J. S. (2006). Involvement of nitric oxide in biofilm dispersal of *Pseudomonas aeruginosa*. *J. Bacteriol.* 188 7344–7353. 10.1128/JB.00779-06 17050922 PMC1636254

[B26] Baxter A. Mittler R. Suzuki N. (2014). ROS as key players in plant stress signaling. *J. Exp. Bot.* 65 1229–1240. 10.1093/jxb/ert375 24253197

[B27] Baxter C. J. Redestig H. Schauer N. Repsilber D. Patil K. R. Nielsen J. (2007). The metabolic response of heterotrophic *Arabidopsis* cells to oxidative stress. *Plant Physiol.* 143 312–325. 10.1104/pp.106.090431 17122072 PMC1761969

[B28] Belhaj K. Lin B. Mauch F. (2009). The chloroplast protein RPH1 plays a role in the immune response of *Arabidopsis* to *Phytophthora brassicae*. *Plant J.* 58 287–298. 10.1111/j.1365-313X.2008.03779.x 19170932

[B29] Beligni M. V. Lamattina L. (1999). Nitric oxide protects against cellular damage produced by methylviologen herbicides in potato plants. *Nitric Oxide* 3 199–208. 10.1006/niox.1999.0222 10442851

[B30] Bell E. Creelman R. A. Mullet J. E. (1995). A chloroplast lipoxygenase is required for wound-induced jasmonic acid accumulation in *Arabidopsis*. *Proc. Natl. Acad. Sci. U.S.A.* 92 8675–8679. 10.1073/pnas.92.19.8675 7567995 PMC41029

[B31] Berridge M. J. Lipp P. Bootman M. D. (2000). The versatility and universality of calcium signaling. *Nat. Rev. Mol. Cell Biol.* 1 11–21. 10.1038/35036191 11413485

[B32] Besson-Bard A. Pugin A. Wendehenne D. (2008). New insights into nitric oxide signaling in plants. *Annu. Rev. Plant Biol.* 59 21–39. 10.1146/annurev.arplant.59.032607.092830 18031216

[B33] Bhattacharjee S. (2005). Reactive oxygen species and oxidative burst: roles in stress, senescence and signal transduction in plants. *Curr. Sci.* 89 1113–1121.

[B34] Bienert G. P. Moller A. L. Kristiansen K. A. Schulz A. Moller I. M. Schjoerring J. K. (2007). Specific aquaporins facilitate the diffusion of hydrogen peroxide across membranes. *J. Biol. Chem.* 282 1183–1192. 10.1074/jbc.M603761200 17105724

[B35] Binder K. A. Wegner L. H. Heidecker M. Zimmermann U. (2003). Gating of Cl-currents in protoplasts from the marine alga *Valonia utricularis* depends on the transmembrane Cl-gradient and is affected by enzymatic cell wall degradation. *J. Membr. Biol.* 191 165–178. 10.1007/s00232-002-1052-2 12571751

[B36] Bird A. (2007). Perceptions of epigenetics. *Nature* 447 396–398. 10.1038/nature05913 17522671

[B37] Bollman K. M. Aukerman M. J. Park M.-Y. Hunter C. Berardini T. Z. Poethig R. S. (2003). HASTY, the *Arabidopsis ortholog* of exportin 5/MSN5, regulates phase change and morphogenesis. *Development* 130 1493–1504. 10.1242/dev.00362 12620976

[B38] Borisova M. M. Kozuleva M. A. Rudenko N. N. Naydov I. A. Klenina I. B. Ivanov B. N. (2012). Photosynthetic electron flow to oxygen and diffusion of hydrogen peroxide through the chloroplast envelope via aquaporins. *Biochim. Biophys. Acta* 1817 1314–1321. 10.1016/j.bbabio.2012.02.036 22421105

[B39] Bouche N. Yellin A. Snedden W. A. Fromm H. (2005). Plant-specific calmodulin-binding proteins. *Annu. Rev. Plant Biol.* 56 435–466. 10.1146/annurev.arplant.56.032604.144224 15862103

[B40] Boveris A. Costa L. E. Poderoso J. J. Carreras M. C. Cadenas E. (2000). Regulation of mitochondrial respiration by oxygen and nitric oxide. *Annu. N. Y. Acad. Sci.* 899 121–135. 10.1111/j.1749-6632.2000.tb06181.x 10863534

[B41] Bowler C. Fluhr R. (2000). The role of calcium and activated oxygens as signals for controlling cross-tolerance. *Trends Plant Sci.* 5 241–246. 10.1016/S1360-1385(00)01628-9 10838614

[B42] Bowler C. Montagu M. V. Inze D. (1992). Superoxide dismutase and stress tolerance. *Annu. Rev. Plant Physiol. Plant Mol. Biol.* 43 83–116. 10.1146/annurev.pp.43.060192.000503

[B43] Bright J. Desikan R. Hancock J. T. Weir I. S. Neill S. J. (2006). ABA-induced NO generation and stomatal closure in *Arabidopsis* are dependent on H_2_O_2_ synthesis. *Plant J.* 45 113–122. 10.1111/j.1365-313X.2005.02615.x 16367958

[B44] Buchanan B. B. Gruissem W. Jones R. L. (2000). *Biochemistry and Molecular Biology of Plants.* Rockville, MD: American Society of Plant Physiologists.

[B45] Caro A. Puntarulo S. (1998). Nitric oxide decreases superoxide anion generation by microsomes from soybean embryonic axes. *Physiol. Plant.* 104 357–364. 10.1034/j.1399-3054.1998.1040310.x

[B46] Carvalho A. Cunha C. Almeida A. J. Osório N. S. Saraiva M. Teixeira-Coelho M. (2012). The rs5743836 polymorphism in TLR9 confers a population-based increased risk of non-Hodgkin lymphoma. *Genes Immun.* 13 197–201. 10.1038/gene.2011.59 21866115 PMC3876733

[B47] Castells E. Puigdomènech P. Casacuberta J. M. (2006). Regulation of the kinase activity of the MIK GCK-like MAP4K by alternative splicing. *Plant. Mol. Biol.* 61 747–756. 10.1007/s11103-006-0046-3 16897489

[B48] Chaouch S. Queval G. Noctor G. (2012). AtRbohF is a crucial modulator of defense-associated metabolism and a key factor in the interplay between intracellular oxidative stress and pathogenesis responses in *Arabidopsis*. *Plant J.* 69 613–627. 10.1111/j.1365-313X.2011.04816.x 21985584

[B49] Chen W. Provart N. J. Glazebrook J. Katagiri F. Chang H. S. Eulgem T. (2002). Expression profile matrix of *Arabidopsis* transcription factor genes suggests their putative functions in response to environmental stresses. *Plant Cell* 14 559–574. 10.1105/tpc.010410 11910004 PMC150579

[B50] Chinnusamy V. Zhu J. K. (2009). Epigenetic regulation of stress responses in plants. *Curr. Opin. Plant Biol.* 12 133–139. 10.1016/j.pbi.2008.12.006 19179104 PMC3139470

[B51] Chiwocha S. D. Cutler A. J. Abrams S. R. Ambrose S. J. Yang J. Ross A. R. (2005). The etr1-2 mutation in *Arabidopsis thaliana* affects the abscisic acid, auxin, cytokinin and gibberellin metabolic pathways during maintenance of seed dormancy, moist-chilling and germination. *Plant J.* 42 35–48. 10.1111/j.1365-313X.2005.02359.x 15773852

[B52] Choi W. G. Toyota M. Kim S. H. Hilleary R. Gilroy S. (2014). Salt stress-induced Ca^2+^ waves are associated with rapid, long-distance root-to-shoot signaling in plants. *Proc. Natl. Acad. Sci. U.S.A.* 111 6497–6502. 10.1073/pnas.1319955111 24706854 PMC4035928

[B53] Coego A. Ramirez V. Gil M. J. Flors V. Mauch-Mani B. Vera P. (2005). An *Arabidopsis* homeodomain transcription factor, OVEREXPRESSOR OF CATIONIC PEROXIDASE 3, mediates resistance to infection by necrotrophic pathogens. *Plant Cell* 17 2123–2137. 10.1105/tpc.105.032375 15923348 PMC1167556

[B54] Correa-Aragunde N. Foresi N. Lamattina L. (2015). Nitric oxide is a ubiquitous signal for maintaining redox balance in plant cells: regulation of ascorbate peroxidase as a case study. *J. Exp. Bot.* 66 2913–2921. 10.1093/jxb/erv073 25750426

[B55] Cosgrove D. J. Hedrich R. (1991). Stretch-activated chloride, potassium, and calcium channels coexisting in plasma membranes of guard cells of *Vicia faba* L. *Planta* 186 143–153. 10.1007/BF00201510 11538499

[B56] Costa V. Moradas-Ferreira P. (2001). Oxidative stress and signal transduction in *Saccharomyces cerevisiae*: insights into ageing, apoptosis and diseases. *Mol. Aspects Med.* 22 217–246. 10.1016/S0098-2997(01)00012-7 11679167

[B57] Covarrubias A. A. Reyes J. L. (2010). Post-transcriptional gene regulation of salinity and drought responses by plant microRNAs. *Plant Cell Environ.* 33 481–489. 10.1111/j.1365-3040.2009.02048.x 19781008

[B58] Czempinski K. Zimmermann S. Ehrhardt T. Muller-Rober B. (1997). New structure and function in plant K^+^ channels: KCO1, an outward rectifier with a steep Ca^2+^ dependency. *EMBO J.* 16 2565–2575. 10.1093/emboj/16.10.2565 9184204 PMC1169868

[B59] Dalle-Donne I. Rossi R. Colombo G. Giustarini D. Milzani A. (2009). Protein S-glutathionylation: a regulatory device from bacteria to humans. *Trends Biochem. Sci.* 34 85–96. 10.1016/j.tibs.2008.11.002 19135374

[B60] Dangl J. L. Jones J. D. G. (2001). Plant pathogens and integrated defense responses to infection. *Nature* 411 826–833. 10.1038/35081161 11459065

[B61] Das R. Pandey G. K. (2010). Expressional analysis and role of calcium regulated kinases in abiotic stress signaling. *Curr. Genomics* 11 2–13. 10.2174/138920210790217981 20808518 PMC2851112

[B62] D’Autreaux B. Toledano M. B. (2007). ROS as signaling molecules: mechanisms that generate specificity in ROS homeostasis. *Nat. Rev. Mol. Cell Biol.* 8 813–824. 10.1038/nrm2256 17848967

[B63] de Jong A. Yakimova E. Kapchina V. Woltering E. (2002). A critical role for ethylene in hydrogen peroxide release during programmed cell death in tomato suspension cells. *Planta* 214 537–545. 10.1007/s004250100654 11925037

[B64] de Pinto M. C. De Gara L. (2004). Changes in the ascorbate metabolism of apoplastic and symplastic spaces are associated with cell differentiation. *J. Exp. Bot.* 55 2559–2569. 10.1093/jxb/erh253 15475379

[B65] De Smet R. Van de Peer Y. (2012). Redundancy and rewiring of genetic networks following genome-wide duplication events. *Curr. Opin. Plant Biol.* 15 168–176. 10.1016/j.pbi.2012.01.003 22305522

[B66] Del Río L. A. (2015). ROS and RNS in plant physiology: an overview. *J. Exp. Bot.* 66 2827–2837. 10.1093/jxb/erv099 25873662

[B67] Delaunay A. Isnard A. D. Toledano M. B. (2000). H_2_O_2_ sensing through oxidation of the Yap1 transcription factor. *EMBO J.* 19 5157–5166. 10.1093/emboj/19.19.5157 11013218 PMC302088

[B68] Delledonne M. Xia Y. Dixon R. A. Lamb C. (1998). Nitric oxide functions as a signal in plant disease resistance. *Nature* 394 585–588. 10.1038/29087 9707120

[B69] Delledonne M. Zeier J. Marocco A. Lamb C. (2001). Signal interactions between nitric oxide and reactive oxygen intermediates in the plant hypersensitive disease resistance response. *Proc. Natl. Acad. Sci. U.S.A.* 98 13454–13459. 10.1073/pnas.231178298 11606758 PMC60892

[B70] Denness L. McKenna J. F. Segonzac C. Wormit A. Madhou P. Bennett M. (2011). Cell wall damage-induced lignin biosynthesis is regulated by a reactive oxygen species- and jasmonic acid-dependent process in *Arabidopsis*. *Plant Physiol.* 156 1364–1374. 10.1104/pp.111.175737 21546454 PMC3135913

[B71] Desikan R. Hancock J. T. Bright J. Harrison J. Weir I. Hooley R. (2005). A role for ETR1 in hydrogen peroxide signaling in stomatal guard cells. *Plant Physiol.* 137 831–834. 10.1104/pp.104.056994 15761208 PMC1065383

[B72] Desikan R. Hancock J. T. Ichimura K. Shinozaki K. Neill S. J. (2001). Harpin induces activation of the *Arabidopsis* mitogen-activated protein kinases AtMPK4 and AtMPK6. *Plant Physiol.* 126 1579–1587. 10.1104/pp.126.4.1579 11500556 PMC117157

[B73] Dietz K. J. (2003). Redox control, redox signaling, and redox homeostasis in plant cells. *Int. Rev. Cytol.* 228 141–193. 10.1016/S0074-7696(03)28004-9 14667044

[B74] Dietz K. J. (2008). Redox signal integration: from stimulus to networks and genes. *Physiol. Plant.* 133 459–468. 10.1111/j.1399-3054.2008.01120.x 18429942

[B75] Dietz K. J. Jacquot J. P. Harris G. (2010). Hubs and bottlenecks in plant molecular signaling networks. *New Phytol.* 188 919–938. 10.1111/j.1469-8137.2010.03502.x 20958306

[B76] Ding J. Li X. Hu H. (2015). MicroRNA modules prefer to bind weak and unconventional target sites. *Bioinformatics* 31 1366–1374. 10.1093/bioinformatics/btu833 25527098 PMC4410656

[B77] Ding Y. Tao Y. Zhu C. (2013). Emerging roles of microRNAs in the mediation of drought stress response in plants. *J. Exp. Bot.* 64 3077–3086. 10.1093/jxb/ert164 23814278

[B78] Dodd A. N. Kudla J. Sanders D. (2010). The language of calcium signaling. *Annu. Rev. Plant Biol.* 61 593–620. 10.1146/annurev-arplant-070109-104628 20192754

[B79] Drerup M. M. Schlücking K. Hashimoto K. Manishankar P. Steinhorst L. Kuchitsu K. (2013). The Calcineurin B-like calcium sensors CBL1 and CBL9 together with their interacting protein kinase CIPK26 regulate the *Arabidopsis* NADPH oxidase RBOHF. *Mol. Plant.* 6 559–569. 10.1093/mp/sst009 23335733

[B80] Dubiella U. Seybold H. Durian G. Komander E. Lassig R. Witte C. P. (2013). Calcium-dependent protein kinase/NADPH oxidase activation circuit is required for rapid defense signal propagation. *Proc. Natl. Acad. Sci. U.S.A.* 110 8744–8749. 10.1073/pnas.1221294110 23650383 PMC3666735

[B81] Durner J. Gow A. J. Stamler J. S. Glazebrook J. (1999). Ancient origins of nitric oxide signaling in biological systems. *Proc. Natl. Acad. Sci. U.S.A.* 96 14206–14207. 10.1073/pnas.96.25.14206 10588683 PMC33950

[B82] Durner J. Wendehenne D. Klessig D. F. (1998). Defense gene induction in tobacco by nitric oxide, cyclic GMP, and cyclic ADP-ribose. *Proc. Natl. Acad. Sci. U.S.A.* 95 10328–10333. 10.1073/pnas.95.17.10328 9707647 PMC21508

[B83] Dynowski M. Schaaf G. Loque D. Moran O. Ludewig U. (2008). Plant plasma membrane water channels conduct the signaling molecule H_2_O_2_. *Biochem. J.* 414 53–61. 10.1042/BJ20080287 18462192

[B84] Ermolayeva E. Sanders D. Johannes E. (1997). Ionic mechanism and role of phytochrome-mediated membrane depolarisation in caulonemal side branch initial formation in the moss Physcomitrella patens. *Planta* 201 109–118. 10.1007/BF01007695

[B85] Evans N. H. McAinsh M. R. Hetherington A. M. Knight M. R. (2005). ROS perception in *Arabidopsis thaliana*: the ozone-induced calcium response. *Plant J.* 41 615–626. 10.1111/j.1365-313X.2004.02325.x 15686524

[B86] Farmer E. E. Ryan C. A. (1992). Octadecanoid precursors of jasmonic acid activate the synthesis of wound-inducible proteinase inhibitors. *Plant Cell* 4 129–134. 10.2307/3869566 12297644 PMC160114

[B87] Fey V. Wagner R. Brautigam K. Wirtz M. Hell R. Dietzmann A. (2005). Retrograde plastid redox signals in the expression of nuclear genes for chloroplast proteins of *Arabidopsis thaliana*. *J. Biol. Chem.* 280 5318–5328. 10.1074/jbc.M406358200 15561727

[B88] Finka A. Cuendet A. F. Maathuis F. J. Saidi Y. Goloubinoff P. (2012). Plasma membrane cyclic nucleotide gated calcium channels control land plant thermal sensing and acquired thermotolerance. *Plant Cell* 24 3333–3348. 10.1105/tpc.112.095844 22904147 PMC3462635

[B89] Foyer C. H. Noctor G. (2005). Redox homeostasis and antioxidant signaling: a metabolic interface between stress perception and physiological responses. *Plant Cell* 17 1866–1875. 10.1105/tpc.105.033589 15987996 PMC1167537

[B90] Foyer C. H. Noctor G. (2009). Redox regulation in photosynthetic organisms: signaling, acclimation, and practical implications. *Antioxid. Redox Signal.* 11 861–905. 10.1089/ars.2008.2177 19239350

[B91] Foyer C. H. Noctor G. (2012). Managing the cellular redox hub in photosynthetic organisms. *Plant Cell Environ.* 35 199–201. 10.1111/j.1365-3040.2011.02453.x 22070467

[B92] Fryer M. J. Ball L. Oxborough K. Karpinski S. Mullineaux P. M. Baker N. R. (2003). Control of Ascorbate Peroxidase 2 expression by hydrogen peroxide and leaf water status during excess light stress reveals a functional organisation of *Arabidopsis* leaves. *Plant J.* 33 691–705. 10.1046/j.1365-313X.2003.01656.x 12609042

[B93] Fujiki Y. Ito M. Nishida I. Watanabe A. (2000). Multiple signaling pathways in gene expression during sugar starvation. Pharmacological analysis of din gene expression in suspension-cultured cells of *Arabidopsis*. *Plant Physiol.* 124 1139–1148. 10.1104/pp.124.3.1139 11080291 PMC59213

[B94] Furbank R. T. Badger M. R. Osmond C. B. (1983). Photoreduction of oxygen in methophyll chloroplast of C4 plants. *Plant Physiol.* 37 1038–1041. 10.1104/pp.73.4.1038 16663325 PMC1066603

[B95] Furchgott R. F. (1995). Special topic: nitric oxide. *Annu. Rev. Physiol.* 57 695–782.

[B96] Gadjev I. Vanderauwera S. Gechev T. S. Laloi C. Minkov I. N. Shulaev V. (2006). Transcriptomic footprints disclose specificity of reactive oxygen species signaling in *Arabidopsis*. *Plant Physiol.* 141 436–445. 10.1104/pp.106.078717 16603662 PMC1475436

[B97] Gasch A. Spellman P. Kao C. Harel O. Eisen M. Storz G. (2000). Genomic expression programs in the response of yeast cells to environmental changes. *Mol. Biol. Cell* 11 4241–4257. 10.1091/mbc.11.12.4241 11102521 PMC15070

[B98] Georgiou G. (2002). How to Flip the (Redox) Switch. *Cell* 111 607–610. 10.1016/S0092-8674(02)01165-0 12464172

[B99] Gill S. S. Tuteja N. (2010). Reactive oxygen species and antioxidant machinery in abiotic stress tolerance in crop plants. *Plant Physiol. Biochem.* 48 909–930. 10.1016/j.plaphy.2010.08.016 20870416

[B100] Glatz A. Vass I. Los D. A. Vigh L. (1999). The Synechocystis model of stress: from molecular chaperonesto membranes. *Plant Physiol. Biochem.* 37 1–12. 10.1016/S0981-9428(99)80061-8

[B101] Glazebrook J. Chen W. Estes B. Chang H. S. Nawrath C. Metraux J. P. (2003). Topology of the network integrating salicylate and jasmonate signal transduction derived from global expression phenotyping. *Plant J.* 34 217–228. 10.1046/j.1365-313X.2003.01717.x 12694596

[B102] Go Y. M. Jones D. P. (2010). Cysteine/cystine redox signaling in cardiovascular disease. *Free Radic. Biol. Med.* 50 495–509. 10.1016/j.freeradbiomed.2010.11.029 21130865 PMC3040416

[B103] Godoy Herz M. A. Kornblihtt A. R. Barta A. Kalyna M. Petrillo E. (2014). Shedding light on the chloroplast as a remote control of nuclear gene expression. *Plant Signal. Behav.* 9:e976150 10.4161/15592324.2014.976150 PMC4622676 25482785

[B104] Goulet A. C. Goldmacher V. S. Lambert J. M. Baron C. Roy D. C. Kouassi E. (1997). Conjugation of blocked ricin to an anti-CD19 monoclonal antibody increases antibody-induced cell calcium mobilization and CD19 internalization. *Blood* 90 2364–2375. 9310487

[B105] Gu Z. Steinmetz L. M. Gu X. Scharfe C. Davis R. W. Li W.-H. (2003). Role of duplicate genes in genetic robustness against null mutations. *Nature* 421 63–66. 10.1038/nature01198 12511954

[B106] Guo Y. Xiong L. Ishitani M. Zhu J. K. (2002). An *Arabidopsis* mutation in translation elongation factor 2 causes superinduction of CBF/DREB1 transcription factor genes but blocks the induction of their downstream targets under low temperatures. *Proc. Natl. Acad. Sci. U.S.A.* 99 7786–7791. 10.1073/pnas.112040099 12032361 PMC124352

[B107] Gustin M. C. Albertyn J. Alexander M. Davenport K. (1998). MAP kinase pathways in the yeast *Saccharomyces cerevisiae*. *Microbiol. Mol. Biol. Rev.* 62 1264–1300. 9841672 10.1128/mmbr.62.4.1264-1300.1998 PMC98946

[B108] Gutkind J. S. (2000). Regulation of mitogen-activated protein kinase signaling networks by G protein-coupled receptors. *Sci STKE* 2000:re1. 10.1126/stke.2000.40.re1 11752597

[B109] Halliwell B. (1991). Reactive oxygen species in living systems: source, biochemistry, and role in human disease. *Am. J. Med.* 91 14S–22S. 10.1016/0002-9343(91)90279-7 1928205

[B110] Halliwell B. (2006). Reactive species and antioxidants. Redox biology is a fundamental theme of aerobic life. *Plant Physiol.* 141 312–322. 10.1104/pp.106.077073 16760481 PMC1475431

[B111] Halliwell B. Gutteridge J. M. C. (1999). *Free Radicals in Biology and Medicine.* Oxford: Oxford University Press.

[B112] Han Y. Chaouch S. Mhamdi A. Queval G. Zechmann B. Noctor G. (2013a). Functional analysis of *Arabidopsis* mutants points to novel roles for glutathione in coupling H_2_O_2_ to activation of salicylic acid accumulation and signaling. *Antioxid. Redox. Signal.* 18 2106–2121. 10.1089/ars.2012.5052 23148658 PMC3629853

[B113] Han Y. Mhamdi A. Chaouch S. Noctor G. (2013b). Regulation of basal and oxidative stress-triggered jasmonic acid-related gene expression by glutathione. *Plant Cell Environ.* 36 1135–1146. 10.1111/pce.12048 23210597

[B114] Hausladen A. Gow A. J. Stamler J. S. (1998). Nitrosative stress: metabolic pathway involving the flavohemoglobin. *Proc. Natl. Acad. Sci. U.S.A.* 95 14100–14105. 10.1073/pnas.95.24.14100 9826660 PMC24333

[B115] Heller J. Tudzynski P. (2011). Reactive oxygen species in phytopathogenic fungi: signaling, development, and disease. *Annu. Rev. Phytopathol.* 49 369–390. 10.1146/annurev-phyto-072910-095355 21568704

[B116] Horvath I. Glatz A. Varvasovszki V. Torok Z. Pali T. Balogh G. (1998). Membrane physical state controls the signaling mechanism of the heat shock response in Synechocystis PCC 6803: identification of hsp17 as a ”fluidity gene”. *Proc. Natl. Acad. Sci. U.S.A.* 95 3513–3518. 10.1073/pnas.95.7.3513 9520397 PMC19867

[B117] Hossain M. A. Bhattacharjee S. Armin S. M. Qian P. Xin W. Li H. Y. (2015). Hydrogen peroxide priming modulates abiotic oxidative stress tolerance: insights from ROS detoxification and scavenging. *Front. Plant Sci.* 6:420 10.3389/fpls.2015.00420 PMC4468828 26136756

[B118] Hu X. Jiang M. Zhang J. Zhang A. Lin F. Tan M. (2007). Calcium-calmodulin is required for abscisic acid-induced antioxidant defense and functions both upstream and downstream of H_2_O_2_ production in leaves of maize (*Zea mays*) plants. *New Phytol.* 173 27–38. 10.1088/1367-2630/9/2/027 17176391

[B119] Hu X. Zhang A. Zhang J. Jiang M. (2006). Abscisic acid is a key inducer of hydrogen peroxide production in leaves of maize plants exposed to water stress. *Plant Cell Physiol.* 47 1484–1495. 10.1093/pcp/pcl014 16990290

[B120] Hufton C. A. Besford R. T. Wellburn A. R. (1996). Effects of NO (+ NO_2_) pollution on growth, nitrate reductase activities and associated protein contents in glasshouse lettuce grown hydroponically in winter with CO_2_ enrichment. *New Phytol.* 133 495–501. 10.1111/j.1469-8137.1996.tb01917.x

[B121] Huner N. P. A. Maxwell D. P. Gray G. R. Savitch L. V. Krol M. Ivanov A. G. (1996). Sensing environmental temperature change through imbalances between energy supply and energy consumption: redox state of photosystem II. *Physiol. Plant.* 98 358–364. 10.1034/j.1399-3054.1996.980218.x

[B122] Ichimura K. Shinozaki K. Tena G. Sheen J. Henry Y. Champion A. (2002). Mitogen-activated protein kinase cascades in plants: a new nomenclature. *Trends Plant Sci.* 7 301–308. 10.1016/S1360-1385(02)02302-6 12119167

[B123] Imlay J. A. (2003). Pathways of oxidative damage. *Annu. Rev. Microbiol.* 57 395–418. 10.1146/annurev.micro.57.030502.090938 14527285

[B124] Jaiswal J. (2001). Calcium - how and why? *J. Biosci.* 26 357–363. 10.1007/BF02703745 11568481

[B125] Jasid S. Simontacchi M. Puntarulo S. (2008). Exposure to nitric oxide protects against oxidative damage but increases the labile iron pool in sorghum embryonic axes. *J. Exp. Bot.* 59 3953–3962. 10.1093/jxb/ern235 18832188 PMC2576640

[B126] Joo J. H. Wang S. Chen J. G. Jones A. M. Fedoroff N. V. (2005). Different signaling and cell death roles of heterotrimeric G protein α and β subunits in the *Arabidopsis* oxidative stress response to ozone. *Plant Cell* 17 957–970. 10.1105/tpc.104.029603 15705948 PMC1069711

[B127] Kadota Y. Sklenar J. Derbyshire P. Stransfeld L. Asai S. Ntoukakis V. (2014). Direct regulation of the NADPH Oxidase RBOHD by the PRR-associated kinase BIK1 during plant immunity. *Mol. Cell.* 54 43–55. 10.1016/j.molcel.2014.02.021 24630626

[B128] Kazan K. (2003). Alternative splicing and proteome diversity in plants: the tip of the iceberg has just emerged. *Trends Plant Sci.* 8 468–471. 10.1016/j.tplants.2003.09.001 14557042

[B129] Khokon A. R. Okuma E. Hossain M. A. Munemasa S. Uraji M. Nakamura Y. (2011). Involvement of extracellular oxidative burst in salicylic acid-induced stomatal closure in *Arabidopsis*. *Plant Cell Environ.* 34 434–443. 10.1111/j.1365-3040.2010.02253.x 21062318

[B130] Khraiwesh B. Zhu J. K. Zhu J. (2012). Role of miRNAs and siRNAs in biotic and abiotic stress responses of plants. *Biochim. Biophys. Acta* 1819 137–148. 10.1016/j.bbagrm.2011.05.001 21605713 PMC3175014

[B131] Kimura S. Kaya H. Kawarazaki T. Hiraoka G. Senzaki E. Michikawa M. (2012). Protein phosphorylation is a prerequisite for the Ca^2+^-dependent activation of *Arabidopsis* NADPH oxidases and may function as a trigger for the positive feedback regulation of Ca^2+^ and reactive oxygen species. *Biochim. Biophys. Acta.* 1823 398–405. 10.1016/j.bbamcr.2011.09.011 22001402

[B132] Klepper L. (1979). Nitric oxide (NO) and nitrogen dioxide (NO_2_) emissions from herbicide-treated soybean plants. *Atmos Environ.* 13 537–542. 10.1016/0004-6981(79)90148-3

[B133] Knight H. Knight M. R. (2001). Abiotic stress signaling pathways: specificity and cross-talk. *Trends Plant Sci.* 6 262–267. 10.1016/S1360-1385(01)01946-X 11378468

[B134] Kochevar I. E. (2004). Singlet oxygen signaling: from intimate to global. *Sci STKE* 2004:e7. 10.1126/stke.2212004pe7 14983102

[B135] Königshofer H. Tromballa H. W. Loppert H. G. (2008). Early events in signaling high-temperature stress in tobacco BY2 cells involve alterations in membrane fluidity and enhanced hydrogen peroxide production. *Plant Cell Environ.* 31 1771–1780. 10.1111/j.1365-3040.2008.01880.x 18761700

[B136] Kornblihtt A. R. (2005). Promoter usage and alternative splicing. *Curr. Opin. Cell Biol.* 17 262–268. 10.1016/j.ceb.2005.04.014 15901495

[B137] Kotchoni S. O. Gachomo E. W. (2006). The reactive oxygen species network pathways: an essential prerequisite for perception of pathogen attack and disease resistance in plants. *J. Biosci.* 31 389–404. 10.1007/BF02704112 17006022

[B138] Koussevitzky S. Nott A. Mockler T. C. Hong F. Sachetto-Martins G. Surpin M. (2007). Signals from chloroplasts converge to regulate nuclear gene expression. *Science* 316 715–719. 10.1126/science.%201140516 17395793

[B139] Kovtun Y. Chiu W. L. Tena G. Sheen J. (2000). Functional analysis of oxidative stress-activated MAPK cascade in plants. *Proc. Natl. Acad. Sci. U.S.A.* 97 2940–2945. 10.1073/pnas.97.6.2940 10717008 PMC16034

[B140] Krasensky J. Jonak C. (2012). Drought salt, and temperature stress-induced metabolic rearrangement and regulatory networks. *J. Exp. Bot.* 4 1593–1608. 10.1093/jxb/err460 22291134 PMC4359903

[B141] Kromer S. (1995). Respiration during photosynthesis. *Annu. Rev. Plant Physiol. Plant Mol. Biol.* 46 45–70. 10.1146/annurev.pp.46.060195.000401

[B142] Kwak J. M. Mori I. C. Pei Z. M. Leonhardt N. Torres M. A. Dangl J. L. (2003). NADPH oxidase AtrbohD and AtrbohF genes function in ROS-dependent ABA signaling in *Arabidopsis*. *EMBO J.* 22 2623–2633. 10.1093/emboj/cdg277 12773379 PMC156772

[B143] Kyriakis J. M. Avruch J. (1996). Sounding the alarm: protein kinase cascades activated by stress and inflammation. *J. Biol. Chem.* 271 24313–24316. 10.1074/jbc.271.40.24313 8798679

[B144] Laloi C. Apel K. Danon A. (2004). Reactive oxygen signaling: the latest news. *Curr. Opin. Plant Biol.* 7 323–328. 10.1016/j.pbi.2004.03.005 15134754

[B145] Lamb C. Dixon R. A. (1997). The oxidative burst in plant disease resistance. *Annu. Rev. Plant Physiol. Plant. Mol. Biol.* 48 251–275. 10.1146/annurev.arplant.48.1.251 15012264

[B146] Leon-Reyes A. Spoel S. H. De Lange E. S. Abe H. Kobayashi M. Tsuda S. (2009). Ethylene modulates the role of NONEXPRESSOR OF PATHOGENESIS-RELATED GENES1 in cross talk between salicylate and jasmonate signaling. *Plant Physiol.* 149 1797–1809. 10.1104/pp.108.133926 19176718 PMC2663751

[B147] Leshem Y. Y. Haramaty E. (1996). The characterization and contrasting effects of the nitric oxide free radical in vegetative stress and senescence of *Pisum sativum* Linn. Foliage. *J. Plant Physiol.* 148 258–263. 10.1016/S0176-1617(96)80251-3

[B148] Leung J. Giraudat J. (1998). Abscisic acid signal transduction. *Annu. Rev. Plant Physiol. Plant Mol. Biol.* 49 199–222. 10.1146/annurev.arplant.49.1.199 15012233

[B149] Levine A. Tenhaken R. Dixon R. Lamb C. (1994). H_2_O_2_ from the oxidative burst orchestrates the plant hypersensitive disease resistance response. *Cell* 79 583–593. 10.1016/0092-8674(94)90544-4 7954825

[B150] Levitt J. (1972). *Responses of Plants to Environmental Stresses.* New York, NY: Academic Press.

[B151] Li H. Shen J. J. Zheng Z. L. Lin Y. Yang Z. (2001). The Rop GTPase switch controls multiple developmental processes in *Arabidopsis*. *Plant Physiol.* 126 670–684. 10.1104/pp.126.2.670 11402196 PMC111158

[B152] Li T. Li H. Zhang Y. X. Liu J. Y. (2011). Identification and analysis of seven H2O2-responsive miRNAs and 32 new miRNAs in the seedlings of rice (*Oryza sativa* L. ssp. indica). *Nucleic Acids Res.* 39 2821–2833. 10.1093/nar/gkq1047 21113019 PMC3074118

[B153] Liu J. Coaker G. (2008). Nuclear trafficking during plant innate immunity. *Mol. Plant* 1 411–422. 10.1093/mp/ssn010 19825550

[B154] Lukosz M. Jakob S. Büchner N. Zschauer T. C. Altschmied J. Haendeler J. (2010). Nuclear redox signaling. *Antioxid. Redox Signal* 12 713–742. 10.1089/ars.2009.2609 19737086

[B155] Lund S. T. Stall R. E. Klee H. J. (1998). Ethylene regulates the susceptible response to pathogen infection in tomato. *Plant Cell* 10 371–382. 10.1105/tpc.10.3.371 9501111 PMC144005

[B156] Ma L. Zhang H. Sun L. Jiao Y. Zhang G. Miao C. (2012). NADPH oxidase AtrbohD and AtrbohF function in ROS-dependent regulation of Na^+^/K^+^ homeostasis in *Arabidopsis* under salt stress. *J. Exp. Bot.* 63 305–317. 10.1093/jxb/err280 21984648

[B157] Madlung A. Comai L. (2004). The effect of stress on genome regulation and structure. *Ann. Bot.* 94 481–495. 10.1093/aob/mch172 15319229 PMC4242226

[B158] Mandadi K. K. Pyle J. D. Scholthof K. G. (2015). Characterization of SCL33 splicing patterns during diverse virus infections in *Brachypodium distachyon*. *Plant Signal. Behav.* 10:e1042641 10.1080/15592324.2015.1042641 PMC4623009 26179847

[B159] Marinho H. S. Real C. Cyrne L. Soares H. Antunes F. (2014). Hydrogen peroxide sensing, signaling and regulation of transcription factors. *Redox Biol.* 2 35–62. 10.1016/j.redox.2014.02.006 PMC3953959 24634836

[B160] Maruta N. Trusov Y. Brenya E. Parekh U. Botella J. R. (2015). Membrane-localized extra-large G proteins and Gbg of the heterotrimeric G proteins form functional complexes engaged in plant immunity in *Arabidopsis*. *Plant Physiol.* 167 1004–1016. 10.1104/pp.114.255703 25588736 PMC4348786

[B161] Maruta T. Inoue T. Tamoi M. Yabuta Y. Yoshimura K. Ishikawa T. (2011). *Arabidopsis* NADPH oxidases, AtrbohD and AtrbohF, are essential for jasmonic acid-induced expression of genes regulated by MYC2 transcription factor. *Plant Sci.* 180 655–660. 10.1016/j.plantsci.2011.01.014 21421415

[B162] Mauch-Mani B. Mauch F. (2005). The role of abscisic acid in plant-pathogen interactions. *Curr. Opin. Plant Biol.* 8 409–414. 10.1016/j.pbi.2005.05.015 15939661

[B163] Maxwell D. P. Wang Y. McIntosh L. (1999). The alternative oxidase lowers mitochondrial reactive oxygen production in plant cells. *Proc. Natl. Acad. Sci. U.S.A.* 96 8271–8276. 10.1073/pnas.96.14.8271 10393984 PMC22224

[B164] McAinsh M. R. Pittman J. K. (2009). Shaping the calcium signature. *New Phytol.* 181 275–294. 10.1111/j.1469-8137.2008.02682.x 19121028

[B165] McCormack E. Tsai Y. C. Braam J. (2005). Handling calcium signaling: *Arabidopsis* CaMs and CMLs. *Trends Plant Sci.* 10 383–389. 10.1016/j.tplants.2005.07.001 16023399

[B166] Merkle T. (2004). Nucleo-cytoplasmic partitioning of proteins in plants: implications for the regulation of environmental and developmental signaling. *Curr. Genet.* 44 231–260. 10.1007/s00294-003-0444-x 14523572

[B167] Mersmann S. Bourdais G. Rietz S. Robatzek S. (2010). Ethylene signaling regulates accumulation of the fls2 receptor and is required for the oxidative burst contributing to plant immunity. *Plant Physiol.* 54 391–400. 10.1104/pp.110.154567 20592040 PMC2938167

[B168] Mhamdi A. Hager J. Chaouch S. Queval G. Han Y. Taconnat L. (2010). *Arabidopsis* GLUTATHIONE REDUCTASE1 plays a crucial role in leaf responses to intracellular hydrogen peroxide and in ensuring appropriate gene expression through both salicylic acid and jasmonic acid signaling pathways. *Plant Physiol.* 153 1144–1160. 10.1104/pp.110.153767 20488891 PMC2899936

[B169] Miller G. Schlauch K. Tam R. Cortes D. Torres M. A. Shulaev V. (2009). The plant NADPH oxidase RBOHD mediates rapid systemic signaling in response to diverse stimuli. *Sci. Signal.* 2:ra45 10.1126/scisignal.2000448 19690331

[B170] Miller G. Suzuki N. Ciftci-Yilmaz S. Mittler R. (2010). Reactive oxygen species homeostasis and signaling during drought and salinity stresses. *Plant Cell Environ.* 33 453–467. 10.1111/j.1365-3040.2009.02041.x 19712065

[B171] Mirouze M. Paszkowski J. (2011). Epigenetic contribution to stress adaptation in plants. *Curr. Opin. Plant Biol.* 14 267–274. 10.1016/j.pbi.2011.03.004 21450514

[B172] Mittler R. (2002). Oxidative stress, antioxidants and stress tolerance. *Trends Plant Sci.* 7 405–410. 10.1016/S1360-1385(02)02312-9 12234732

[B173] Mittler R. Vanderauwera S. Gollery M. Van Breusegem F. (2004). Reactive oxygen gene network of plants. *Trends Plant Sci.* 9 490–498. 10.1016/j.tplants.2004.08.009 15465684

[B174] Mittler R. Vanderauwera S. Suzuki N. Miller G. Tognetti V. B. Vandepoele K. (2011). ROS signaling: the new wave? *Trends Plant Sci.* 16 300–309. 10.1016/j.tplants.2011.03.007 21482172

[B175] Miura K. Okamoto H. Okuma E. Shiba H. Kamada H. Hasegawa P. M. (2013). SIZ1 deficiency causes reduced stomatal aperture and enhanced drought tolerance via controlling salicylic acid-induced accumulation of reactive oxygen species in *Arabidopsis*. *Plant J.* 73 91–104. 10.1111/tpj.12014 22963672

[B176] Moan J. (1990). On the diffusion length of singlet oxygen in cells and tissues. *J. Photochem. Photobiol.* 6 343–344. 10.1016/1011-1344(90)85104-5

[B177] Molassiotis A. Fotopoulos V. (2011). Oxidative and nitrosative signaling in plants: two branches in the same tree? *Plant Signal. Behav.* 6 210–214. 10.4161/psb.6.2.14878 21325889 PMC3121980

[B178] Møller I. M. Jensen P. E. Hansson A. (2007). Oxidative modifications to cellular components in plants. *Annu. Rev. Plant Biol.* 58 459–481. 10.1146/annurev.arplant.58.032806.103946 17288534

[B179] Moller I. M. Sweetlove L. J. (2010). ROS signaling - specificity is required. *Trends Plant Sci.* 15 370–374. 10.1016/j.tplants.2010.04.008 20605736

[B180] Monshausen G. B. Bibikova T. N. Weisenseel M. H. Gilroy S. (2009). Ca^2+^ regulates reactive oxygen species production and pH during mechanosensing in *Arabidopsis* roots. *Plant Cell* 21 2341–2356. 10.1105/tpc.109.068395 19654264 PMC2751959

[B181] Moreau M. Lindermayr C. Durner J. Klessig D. F. (2010). NO synthesis and signaling in plants–where do we stand? *Physiol. Plant.* 138 372–383. 10.1111/j.1399-3054.2009.01308.x 19912564

[B182] Mubarakshina M. M. Ivanov B. N. Naydov I. A. Hillier W. Badger M. R. Krieger-Liszkay A. (2010). Production and diffusion of chloroplastic H_2_O_2_ and its implication to signaling. *J. Exp. Bot.* 61 3577–3587. 10.1093/jxb/erq171 20595239

[B183] Mühlenbock P. Szechynska-Hebda M. Plaszczyca M. Baudo M. Mateo A. Mullineaux P. M. (2008). Chloroplast signaling and LESION SIMULATING DISEASE1 regulate crosstalk between light acclimation and immunity in *Arabidopsis*. *Plant Cell* 20 2339–2356. 10.1105/tpc.108.059618 18790826 PMC2570729

[B184] Mur L. A. Kenton P. Atzorn R. Miersch O. Wasternack C. (2006). The outcomes of concentration-specific interactions between salicylate and jasmonate signaling include synergy, antagonism, and oxidative stress leading to cell death. *Plant Physiol.* 140 249–262. 10.1104/pp.105.072348 16377744 PMC1326048

[B185] Murata N. Los D. A. (1997). Membrane fluidity and temperature perception. *Plant Physiol.* 115 875–879. 12223851 10.1104/pp.115.3.875 PMC158550

[B186] Nagarathnam B. Kalaimathy S. Balakrishnan V. Sowdhamini R. (2012). Cross-genome clustering of human and C. *elegans* G-Protein Coupled Receptors. *Evol. Bioinform.* 8 229–259. 10.4137/EBO.S9405 PMC3396462 22807621

[B187] Nakagami H. Pitzschke A. Hirt H. (2005). Emerging MAP kinase pathways in plant stress signaling. *Trends Plant Sci.* 10 339–346. 10.1016/j.tplants.2005.05.009 15953753

[B188] Nathan C. (2003). Specificity of a third kind: reactive oxygen and nitrogen intermediates in cell signaling. *J. Clin. Invest.* 111 769–778. 10.1172/JCI200318174 12639979 PMC153776

[B189] Navarro L. Dunoyer P. Jay F. Arnold B. Dharmasiri N. Estelle M. (2006). A plant miRNA contributes to antibacterial resistance by repressing auxin signaling. *Science* 312 436–439. 10.1126/science.1126088 16627744

[B190] Neill S. J. Desikan R. Clarke A. Hurst R. D. Hancock J. T. (2002). Hydrogen peroxide and nitric oxide as signaling molecules in plants. *J. Exp. Bot.* 53 1237–1247. 10.1093/jexbot/53.372.1237 11997372

[B191] Neill S. J. Desikan R. Hancock J. T. (2003). Nitric oxide signaling in plants. *New Phytol.* 159 11–35. 10.1046/j.1469-8137.2003.00804.x 33873677

[B192] Niedre M. Patterson M. S. Wilson B. C. (2002). Direct near-infrared luminescence detection of singlet oxygen generated by photodynamic therapy in cells in vitro and tissues in vivo. *Photochem. Photobiol.* 75 382–391. 10.1562/0031-8655(2002)0750382DNILDO2.0.CO2 12003128

[B193] Noctor G. (1998). Ascorbate and glutathione: keeping active oxygen under control. *Annu. Rev. Plant Physiol. Plant Mol. Biol.* 49 249–279. 10.1146/annurev.arplant.49.1.249 15012235

[B194] Noctor G. (2006). Metabolic signaling in defense and stress: the central roles of soluble redox couples. *Plant Cell Environ.* 29 409–425. 10.1111/j.1365-3040.2005.01476.x 17080595

[B195] Noctor G. Mhamdi A. Foyer C. H. (2014). The roles of reactive oxygen metabolism in drought: not so cut and dried. *Plant Physiol.* 164 1636–1648. 10.1104/pp.113.233478 24715539 PMC3982730

[B196] Nose K. (2005). Redox control of protein trafficking. *Antioxid. Redox Signal.* 7 303–307. 10.1089/ars.2005.7.303 15706078

[B197] Nott A. Jung H. S. Koussevitzky S. Chory J. (2006). Plastid-to-nucleus retrograde signaling. *Annu. Rev. Plant Biol.* 57 739–759. 10.1146/annurev.arplant.57.032905.105310 16669780

[B198] Orozco-Cardenas M. Ryan C. A. (1999). Hydrogen peroxide is generated systemically in plant leaves by wounding and systemin via the octadecanoid pathway. *Proc. Natl. Acad. Sci. U.S.A.* 96 6553–6557. 10.1073/pnas.96.11.6553 10339626 PMC26920

[B199] Orzaez D. Granell A. (1997). DNA fragmentation is regulated by ethylene during carpel senescence in *Pisum sativum*. *Plant J.* 11 137–144. 10.1046/j.1365-313X.1997.11010137.x

[B200] Overmyer K. Tuominen H. Kettunen R. Betz C. Langebartels C. Sandermann H. (2000). Ozone-sensitive *Arabidopsis* rcd1 mutant reveals opposite roles for ethylene and jasmonate signaling pathways in regulating superoxide-dependent cell death. *Plant Cell* 12 1849–1862. 10.1105/tpc.12.10.1849 11041881 PMC149124

[B201] Papin J. A. Hunter T. Palsson B. O. Subramaniam S. (2005). Reconstruction of cellular signaling networks and analysis of their properties. *Nat. Rev. Mol. Cell Biol.* 6 99–111. 10.1038/nrm1570 15654321

[B202] Park S.-W. Kaimoyo E. Kumar D. Mosher S. Klessig D. F. (2007). Methyl salicylate is a critical mobile signal for plant systemic acquired resistance. *Science* 318 113–116. 10.1126/science.1147113 17916738

[B203] Parker J. E. Aarts N. Austin M. A. Feys B. J. Moisan L. J. Muskett P. (2001). Genetic analysis of plant disease resistance pathways. *Novartis Found. Symp.* 236 153–161. 11387977 10.1002/9780470515778.ch11

[B204] Parry G. (2015). The plant nuclear envelope and regulation of gene expression. *J. Exp. Bot.* 66 1673–1685. 10.1093/jxb/erv023 25680795

[B205] Paulsen C. E. Carroll K. S. (2010). Orchestrating redox signaling networks through regulatory cysteine switches. *ACS Chem. Biol.* 5 47–62. 10.1021/cb900258z 19957967 PMC4537063

[B206] Perez I. B. Brown P. J. (2014). The role of ROS signaling in cross-tolerance: from model to crop. *Front Plant Sci.* 5:754 10.3389/fpls.2014.00754 PMC4274871 25566313

[B207] Petrov V. D. Van Breusegem F. (2012). Hydrogen peroxide-a central hub for information flow in plant cells. *AoB Plants* 2012:ls014 10.1093/aobpla/pls014 PMC3366437 22708052

[B208] Pfannschmidt T. Bräutigam K. Wagner R. Dietzel L. Schröter Y. Steiner S. (2009). Potential regulation of gene expression in photosynthetic cells by redox and energy state: approaches towards better understanding. *Ann. Bot.* 103 599–607. 10.1093/aob/mcn081 18492734 PMC2707342

[B209] Pfannschmidt T. Nilsson A. Allen J. F. (1999). Photosynthetic control of chloroplast gene expression. *Nature* 397 625–628. 10.1038/17624

[B210] Phaniendra A. Jestadi D. B. Periyasamy L. (2015). Free radicals: properties, sources, targets, and their implication in various diseases. *Indian J. Clin. Biochem.* 30 11–26. 10.1007/s12291-014-0446-0 25646037 PMC4310837

[B211] Pitzschke A. Hirt H. (2006). Mitogen-activated protein kinases and reactive oxygen species signaling in plants. *Plant Physiol.* 141 351–356. 10.1104/pp.106.079160 16760487 PMC1475449

[B212] Pitzschke A. Schikora A. Hirt H. (2009). MAPK cascade signaling networks in plant defense. *Curr. Opin. Plant Biol.* 12 421–426. 10.1016/j.pbi.2009.06.008 19608449

[B213] Pogson B. J. Woo N. S. Förster B. Small I. D. (2008). Plastid signaling to the nucleus and beyond. *Trends Plant Sci.* 13 602–609. 10.1016/j.tplants.2008.08.008 18838332

[B214] Polidoros A. N. Mylona P. V. Pasentsis K. Scandalios J. G. Tsaftaris A. S. (2005). The maize alternative oxidase 1a (Aox1a) gene is regulated by signals related to oxidative stress. *Redox Rep.* 10 71–78. 10.1179/135100005X21688 15949126

[B215] Pospisil P. Arato A. Krieger-Liszkay A. Rutherford A. W. (2004). Hydroxyl radical generation by photosystem II. *Biochemistry* 43 6783–6792. 10.1021/bi036219i 15157112

[B216] Potters G. Horemans N. Bellone S. Caubergs R. J. Trost P. Guisez Y. (2004). Dehydroascorbate influences the plant cell cycle through a glutathione-independent reduction mechanism. *Plant Physiol.* 134 1479–1487. 10.1104/pp.103.033548 15047900 PMC419824

[B217] Potters G. Horemans N. Jansen M. A. (2010). The cellular redox state in plant stress biology-a charging concept. *Plant Physiol. Biochem.* 48 292–300. 10.1016/j.plaphy.2009.12.007 20137959

[B218] Price A. H. Taylor A. Ripley S. J. Griffiths A. Trewavas A. J. Knight M. R. (1994). Oxidative signals in tobacco increase cytosolic calcium. *Plant Cell* 6 1301–1310. 10.1105/tpc.6.9.1301 12244272 PMC160521

[B219] Qi M. Elion E. A. (2005). MAP kinase pathways. *J. Cell Sci.* 118 3569–3572. 10.1242/jcs.02470 16105880

[B220] Raghavendra A. S. Gonugunta V. K. Christmann A. Grill E. (2010). ABA perception and signaling. *Trends Plant Sci.* 15 395–401. 10.1016/j.tplants.2010.04.006 20493758

[B221] Rao M. V. Lee H. Creelman R. A. Mullet J. E. Davis K. R. (2000). Jasmonic acid signaling modulates ozone-induced hypersensitive cell death. *Plant Cell* 12 1633–1646. 10.1105/tpc.12.9.1633 11006337 PMC149075

[B222] Reddy A. S. N. (2007). Alternative splicing of pre-messenger RNAs in plants in the genomic era. *Annu. Rev. Plant Biol.* 58 267–294. 10.1146/annurev.arplant.58.032806.103754 17222076

[B223] Rentel M. C. Lecourieux D. Ouaked F. Usher S. L. Petersen L. Okamoto H. (2004). OXI1 kinase is necessary for oxidative burst-mediated signaling in *Arabidopsis*. *Nature* 427 858–861. 10.1038/nature02353 14985766

[B224] Riechmann J. L. Heard J. Martin G. Reuber L. Jiang C. Keddie J. (2000). *Arabidopsis* transcription factors: genome-wide comparative analysis among eukaryotes. *Science* 290 2105–2110. 10.1126/science.290.5499.2105 11118137

[B225] Rodriguez M. C. Petersen M. Mundy J. (2010). Mitogen-activated protein kinase signaling in plants. *Annu. Rev. Plant. Biol.* 61 621–649. 10.1146/annurev-arplant-042809-112252 20441529

[B226] Sagi M. Fluhr R. (2006). Production of reactive oxygen species by plant NADPH oxidases. *Plant Physiol.* 141 336–340. 10.1104/pp.106.078089 16760484 PMC1475462

[B227] Sahu P. P. Pandey G. Sharma N. Puranik S. Muthamilarasan M. Prasad M. (2013). Epigenetic mechanisms of plant stress responses and adaptation. *Plant Cell Rep.* 32 1151–1159. 10.1007/s00299-013-1462-x 23719757

[B228] Saidi Y. Finka A. Muriset M. Bromberg Z. Weiss Y. G. Maathuis F. J. (2009). The heat shock response in moss plants is regulated by specific calcium-permeable channels in the plasma membrane. *Plant Cell* 21 2829–2843. 10.1105/tpc.108.065318 19773386 PMC2768932

[B229] Scandalios J. G. (2002). Oxidative stress responses - what have genome-scale studies taught us? *Genome Biol.* 3:REVIEWS1019 10.1186/gb-2002-3-7-reviews1019 PMC139384 12184812

[B230] Scandalios J. G. (2005). Oxidative stress: molecular perception and transduction of signals triggering antioxidant gene defenses. *Braz. J. Med. Biol. Res.* 38 995–1014. 10.1590/S0100-879X2005000700003 16007271

[B231] Scheibe R. Dietz K. J. (2012). Reduction-oxidation network for flexible adjustment of cellular metabolism in photoautotrophic cells. *Plant Cell Environ.* 35 202–216. 10.1111/j.1365-3040.2011.02319.x 21410714

[B232] Schenk P. M. Kazan K. Wilson I. Anderson J. P. Richmond T. Somerville S. C. (2000). Coordinated plant defense responses in *Arabidopsis* revealed by microarray analysis. *Proc. Natl. Acad. Sci. U.S.A.* 97 11655–11660. 10.1073/pnas.97.21.11655 11027363 PMC17256

[B233] Schmitt F. J. Renger G. Friedrich T. Kreslavksi V. D. Zharmukhadmedov S. K. Los D. A. (2014). Reactive oxygen species: re-evaluation of generation, monitoring and role in stress-signaling in phototrophicorgan- isms. *Biochim. Biophys. Acta* 1837 835–848. 10.1016/j.bbabio.2014.02.005 24530357

[B234] Senaratna T. Touchell D. Bunn E. Dixon K. (2000). Acetyl salicylic acid (Aspirin) and salicylic acid induce multiple stress tolerance in bean and tomato plants. *Plant Growth Regulat.* 30 157–161. 10.1023/A:1006386800974

[B235] Serrato A. J. Fernández-Trijueque J. Barajas-López J. D. Chueca A. Sahrawy M. (2013). Plastid thioredoxins: a “one-for-all” redox-signaling system in plants. *Front. Plant Sci.* 21:463 10.3389/fpls.2013.00463 PMC3836485 24319449

[B236] Sewelam N. Jaspert N. Kelen K. V. D. Schmitz J. Frerigmann H. Tognetti V. B. (2014a). Spatial H_2_O_2_ signaling specificity: H_2_O_2_ from chloroplasts and peroxisomes differentially modulates the plant transcriptome. *Mol. Plant* 7 1191–1210. 10.1093/mp/ssu070 24908268

[B238] Sewelam N. Kazan K. Thomas-Hall S. Kidd B. N. Manners J. M. Schenk P. M. (2013). Ethylene response factor 6 is a regulator of reactive oxygen species signaling in *Arabidopsis*. *PLoS ONE* 8:e70289 10.1371/journal.pone.0070289 PMC3734174 23940555

[B237] Sewelam N. Oshima Y. Mitsuda N. Ohme-Takagi M. (2014b). A step towards understanding plant responses to multiple environmental stresses: a genome-wide study. *Plant Cell Environ.* 37 2024–2035. 24417440 10.1111/pce.12274

[B239] Shen Q. H. Saijo Y. Mauch S. Biskup C. Bieri S. Keller B. (2007). Nuclear activity of MLA immune receptors links isolate-specific and basal disease-resistance responses. *Science* 315 1098–1103. 10.1126/science.1136372 17185563

[B240] Simon-Plas F. Elmayan T. Blein J.-P. (2002). The plasma membrane oxidase NtrbohD is responsible for AOS production in elicited tobacco cells. *Plant J.* 31 137–147. 10.1046/j.1365-313X.2002.01342.x 12121444

[B241] Sinha A. K. Jaggi M. Raghuram B. Tuteja N. (2011). Mitogen-activated protein kinase signaling in plants under abiotic stress. *Plant Signal. Behav.* 6 196–203. 10.4161/psb.6.2.14701 21512321 PMC3121978

[B242] Springer N. M. (2013). Epigenetics and crop improvement. *Trends Genet.* 29 241–247. 10.1016/j.tig.2012.10.009 23128009

[B243] Stael S. Wurzinger B. Mair A. Mehlmer N. Vothknecht U. C. Teige M. (2012). Plant organellar calcium signaling: an emerging field. *J. Exp. Bot.* 63 1525–1542. 10.1093/jxb/err394 22200666 PMC3966264

[B244] Stelling J. Sauer U. Szallasi Z. Doyle F. J. Doyle J. (2004). Robustness of cellular functions. *Cell* 118 675–685. 10.1016/j.cell.2004.09.008 15369668

[B245] Storz G. Imlayt J. A. (1999). Oxidative stress. *Curr. Opin. Microbiol.* 2 188–194. 10.1016/S1369-5274(99)80033-2 10322176

[B246] Storz G. Tartaglia L. A. Ames B. N. (1990). Transcriptional regulator of oxidative stress-inducible genes: direct activation by oxidation. *Science* 248 189–194. 10.1126/science.2183352 2183352

[B247] Suharsono U. Fujisawa Y. Kawasaki T. Iwasaki Y. Satoh H. Shimamoto K. (2002). The heterotrimeric G protein alpha subunit acts upstream of the small GTPase Rac in disease resistance of rice. *Proc. Natl. Acad. Sci. U.S.A.* 99 13307–13312. 10.1073/pnas.192244099 12237405 PMC130629

[B248] Sunkar R. Kapoor A. Zhu J. K. (2006). Posttranscriptional induction of two Cu/Zn superoxide dismutase genes in *Arabidopsis* is mediated by downregulation of miR398 and important for oxidative stress tolerance. *Plant Cell* 18 2051–2065. 10.1105/tpc.106.041673 16861386 PMC1533975

[B249] Suzuki N. Koussevitzky S. Mittler R. Miller G. (2012). ROS and redox signaling in the response of plants to abiotic stress. *Plant Cell Environ.* 35 259–270. 10.1111/j.1365-3040.2011.02336.x 21486305

[B250] Suzuki N. Miller G. Salazar C. Mondal H. A. Shulaev E. Cortes D. F. (2013). Temporal-spatial interaction between reactive oxygen species and abscisic acid regulates rapid systemic acclimation in plants. *Plant Cell* 25 3553–3569. 10.1105/tpc.113.114595 24038652 PMC3809549

[B251] Takahashi F. Mizoguchi T. Yoshida R. Ichimura K. Shinozaki K. (2011). Calmodulin-dependent activation of MAP kinase for ROS homeostasis in *Arabidopsis*. *Mol. Cell* 41 649–660. 10.1016/j.molcel.2011.02.029 21419340

[B252] Tanou G. Job C. Belghazi M. Molassiotis A. Diamantidis G. Job D. (2010). Proteomic signatures uncover hydrogen peroxide and nitric oxide cross-talk signaling network in citrus plants. *J. Proteome Res.* 9 5994–6006. 10.1021/pr100782h 20825250

[B253] Tanou G. Job C. Rajjou L. Arc E. Belghazi M. Diamantidis G. (2009). Proteomics reveals the overlapping roles of hydrogen peroxide and nitric oxide in the acclimation of citrus plants to salinity. *Plant J.* 60 795–804. 10.1111/j.1365-313X.2009.04000.x 19682288

[B254] Tena G. Asai T. Chiu W. L. Sheen J. (2001). Plant mitogen-activated protein kinase signaling cascades. *Curr. Opin. Plant Biol.* 4 392–400. 10.1016/S1369-5266(00)00191-6 11597496

[B255] Thanaraj T. A. (2004). ASD: the alternative splicing database. *Nucleic Acids Res.* 32 D64–D69. 10.1093/nar/gkh030 14681360 PMC308764

[B256] Thomma B. P. Penninckx I. A. Broekaert W. F. Cammue B. P. (2001). The complexity of disease signaling in *Arabidopsis*. *Curr. Opin. Immunol.* 13 63–68. 10.1016/S0952-7915(00)00183-7 11154919

[B257] Tokunaga T. Miyahara K. Tabata K. Esaka M. (2005). Generation and properties of ascorbic acid-overproducing transgenic tobacco cells expressing sense RNA for l-galactono-1,4-lactone dehydrogenase. *Planta* 220 854–863. 10.1007/s00425-004-1406-3 15549373

[B258] Torres M. A. Dangl J. L. (2005). Functions of the respiratory burst oxidase in biotic interactions, abiotic stress and development. *Curr. Opin. Plant Biol.* 8 397–403. 10.1016/j.pbi.2005.05.014 15939662

[B259] Torres M. A. Dangl J. L. Jones J. D. G. (2002). *Arabidopsis* gp91phox homologues AtrbohD and AtrbohF are required for accumulation of reactive oxygen intermediates in the plant defense response. *Proc. Natl. Acad. Sci. U.S.A.* 99 517–522. 10.1073/pnas.012452499 11756663 PMC117592

[B260] Torres M. A. Jones J. D. Dangl J. L. (2005). Pathogen-induced, NADPH oxidase-derived reactive oxygen intermediates suppress spread of cell death in *Arabidopsis thaliana*. *Nat. Genet.* 37 1130–1134. 10.1038/ng1639 16170317

[B261] Torres M. A. Morales J. Sánchez-Rodríguez C. Molina A. Dangl J. L. (2013). Functional interplay between *Arabidopsis* NADPH oxidases and heterotrimeric G protein. *Mol. Plant Microbe Interact.* 26 686–694. 10.1094/MPMI-10-12-0236-R 23441575

[B262] Torres M. A. Onouchi H. Hamada S. Machida C. Hammond-Kosack K. E. Jones J. D. G. (1998). Six *Arabidopsis thaliana* homologues of the human respiratory burst oxidase (gp91phox). *Plant J.* 14 365–370. 10.1046/j.1365-313X.1998.00136.x 9628030

[B263] Triantaphylidès C. Havaux M. (2009). Singlet oxygen in plants: production, detoxification and signaling. *Trends Plant Sci.* 14 219–228. 10.1016/j.tplants.2009.01.008 19303348

[B264] Trusov Y. Sewelam N. Rookes J. E. Kunkel M. Nowak E. Schenk P. M. (2009). Heterotrimeric G proteins-mediated resistance to necrotrophic pathogens includes mechanisms independent of salicylic acid-, jasmonic acid/ethylene- and abscisic acid-mediated defense signaling. *Plant J.* 58 69–81. 10.1111/j.1365-313X.2008.03755.x 19054360

[B265] van Rooij E. (2011). The art of microRNA research. *Circ. Res.* 108 219–234. 10.1161/CIRCRESAHA.110.227496 21252150

[B266] Vanderauwera S. Suzuki N. Miller G. van de Cotte B. Morsa S. Ravanat J. L. (2011). Extranuclear protection of chromosomal DNA from oxidative stress. *Proc. Natl. Acad. Sci. U.S.A.* 108 1711–1716. 10.1073/pnas.1018359108 21220338 PMC3029710

[B267] Vanyushin B. F. Ashapkin V. V. (2011). DNA methylation in higher plants: past, present and future. *Biochim. Biophys. Acta* 1809 360–368. 10.1016/j.bbagrm.2011.04.006 21549230

[B268] Verslues P. E. Zhu J. (2004). Before and beyond ABA: upstream sensing and internal signals that determine ABA accumulation and response under abiotic stress. *Biochem. Soc. Trans.* 33 375–379. 10.1042/BST0330375 15787610

[B269] Vlot A. C. Liu P.-P. Cameron R. K. Park S.-W. Yang Y. Kumar D. (2008). Identification of likely orthologs of tobacco salicylic acid-binding protein 2 and their role in systemic acquired resistance in *Arabidopsis thaliana*. *Plant J.* 56 445–456. 10.1111/j.1365-313X.2008.03618.x 18643994

[B270] Walley J. W. Dehesh K. (2009). Molecular mechanisms regulating rapid stress signaling networks in *Arabidopsis*. *J. Integr. Plant Biol.* 52 354–359. 10.1111/j.1744-7909.2010.00940.x 20377697

[B271] Wang B.-B. Brendel V. (2006). Genomewide comparative analysis of alternative splicing in plants. *Proc. Natl. Acad. Sci. U.S.A.* 103 7175–7180. 10.1073/pnas.0602039103 16632598 PMC1459036

[B273] Wang P. Du Y. Hou Y. J. Zhao Y. Hsu C. C. Yuan F. (2015). Nitric oxide negatively regulates abscisic-acid signaling in guard cells by S-nitrosylation of OST1. *Proc. Natl. Acad. Sci. U.S.A.* 112 613–618. 10.1073/pnas.1423481112 25550508 PMC4299189

[B274] Wang P. Du Y. Li Y. Ren D. Song C. P. (2010). Hydrogen peroxide–mediated activation of MAP kinase 6 modulates nitric oxide biosynthesis and signal transduction in *Arabidopsis*. *Plant Cell* 22 2981–2998. 10.1105/tpc.109.072959 20870959 PMC2965546

[B272] Wang P.-C. Du Y.-Y. An G.-Y. Zhou Y. Miao C. Song C.-P. (2006). Analysis of global expression profiles of *Arabidopsis* genes under abscisic acid and H_2_O_2_ applications. *J. Integ. Plant Biol.* 48 62–74. 10.1111/j.1744-7909.2006.00213.x

[B275] Wasternack C. Hause B. (2013). Jasmonates: biosynthesis, perception, signal transduction and action in plant stress response, growth and development. An update to the 2007 review in Annals of Botany. *Ann. Bot.* 111 1021–1058. 10.1093/aob/mct067 23558912 PMC3662512

[B276] Wi S. J. Ji N. R. Park K. Y. (2012). Synergistic biosynthesis of biphasic ethylene and reactive oxygen species in response to hemibiotrophic *Phytophthora parasitica* in tobacco plants. *Plant Physiol.* 159 251–265. 10.1104/pp.112.194654 22388490 PMC3375963

[B277] Wildt J. Kley D. Rockel A. Rockel P. Segschneider H. J. (1997). Emission of NO from several higher plant species. *J. Geophys. Res.* 102 5919–5927. 10.1029/96JD02968

[B278] Wrzaczek M. Brosché M. Kangasjärvi J. (2013). ROS signaling loops — production, perception, regulation. *Curr. Opin. Plant Biol.* 16 575–582. 10.1016/j.pbi.2013.07.002 23876676

[B279] Wrzaczek M. Hirt H. (2001). Plant MAP kinase pathways: how many and what for? *Biol. Cell* 93 81–87. 10.1016/S0248-4900(01)01121-2 11730326

[B280] Xia X. J. Zhou Y. H. Shi K. Zhou J. Foyer C. H. Yu J. Q. (2015). Interplay between reactive oxygen species and hormones in the control of plant development and stress tolerance. *J. Exp. Bot.* 66 2839–2856. 10.1093/jxb/erv089 25788732

[B281] Xiao Y. Savchenko T. Baidoo E. E. Chehab W. E. Hayden D. M. Tolstikov V. (2012). Retrograde signaling by the plastidial metabolite MEcPP regulates expression of nuclear stress-response genes. *Cell* 149 1525–1535. 10.1016/j.cell.2012.04.038 22726439

[B282] Xing Y. Jia W. Zhang J. (2008). AtMKK1 mediates ABA-induced CAT1 expression and H_2_O_2_ production via AtMPK6-coupled signaling in *Arabidopsis*. *Plant J.* 54 440–451. 10.1111/j.1365-313X.2008.03433.x 18248592

[B283] Xiong L. Schumaker K. S. Zhu J.-K. (2002). Cell Signaling during Cold, Drought, and Salt Stress. *Plant Cell* 14 s165–s183. 12045276 10.1105/tpc.000596 PMC151254

[B284] Xu D. B. Chen M. Ma Y. N. Xu Z. S. Li L. C. Chen Y. F. (2015). A G-protein β subunit, AGB1, negatively regulates the ABA response and drought tolerance by down-regulating AtMPK6-related pathway in *Arabidopsis*. *PLoS ONE* 10:e0116385 10.1371/journal.pone.0116385 PMC4312036 25635681

[B285] Xue H. Seifert G. J. (2015). Fasciclin like arabinogalactan protein 4 and respiratory burst oxidase homolog d and F independently modulate abscisic acid signaling. *Plant Signal. Behav.* 10:e989064 10.4161/15592324.2014.989064 PMC4622830 25826261

[B286] Yoshioka H. Asai S. Yoshioka M. Kobayashi M. (2009). Molecular mechanisms of generation for nitric oxide and reactive oxygen species, and role of the radical burst in plant immunity. *Mol. Cell* 28 321–329. 10.1007/s10059-009-0156-2 19830396

[B287] Yoshioka H. Numata N. Nakajima K. Katou S. Kawakita K. Rowland O. (2003). Nicotiana benthamiana gp91phox homologs NbrbohA and NbrbohB participate in H_2_O_2_ accumulation and resistance to *Phytophthora infestans*. *Plant Cell* 15 706–718. 10.1105/tpc.008680 12615943 PMC150024

[B288] Yuasa T. Ichimura K. Mizoguchi T. Shinozaki K. (2001). Oxidative stress activates ATMPK6, an *Arabidopsis* homologue of MAP kinase. *Plant Cell Physiol.* 42 1012–1016. 10.1093/pcp/pce123 11577197

[B289] Zarembinski T. I. Theologis A. (1994). Ethylene biosynthesis and action: a case of conservation. *Plant Mol. Biol.* 26 1579–1597. 10.1007/BF00016491 7858205

[B290] Zhang L. Cheng Z. Qin R. Qiu Y. Wang J. L. Cui X. (2012). Identification and characterization of an epi-allele of fie1 reveals a regulatory linkage between two epigenetic marks in rice. *Plant Cell* 24 4407–4421. 10.1105/tpc.112.102269 23150632 PMC3531842

[B291] Zhang W. Jeon B. W. Assmann S. M. (2011). Heterotrimeric G-protein regulation of ROS signaling and calcium currents in *Arabidopsis* guard cells. *J. Exp. Bot.* 62 2371–2379. 10.1093/jxb/erq424 21262908

[B292] Zhou J. Wang J. Li X. Xia X. J. Zhou Y. H. Shi K. (2014). H_2_O_2_ mediates the crosstalk of brassinosteroid and abscisic acid in tomato responses to heat and oxidative stresses. *J. Exp. Bot.* 65 4371–4383. 10.1093/jxb/eru217 24899077 PMC4112640

[B293] Zhu Y. Zuo M. Liang Y. Jiang M. Zhang J. Scheller H. V. (2013). MAP65-1a positively regulates H_2_O_2_ amplification and enhances brassinosteroid-induced antioxidant defense in maize. *J. Exp. Bot.* 64 3787–3802. 10.1093/jxb/ert215 23956414 PMC3745737

[B294] Zurbriggen M. D. Carrillo N. Tognetti V. B. Melzer M. Peisker M. Hause B. (2009). Chloroplast-generated reactive oxygen species play a major role in localized cell death during the non-host interaction between tobacco and *Xanthomonas campestris*pv. vesicatoria. *Plant J.* 60 962–973. 10.1111/j.1365-313X.2009.04010.x 19719480

